# Inhibition of transmembrane TNF-α shedding by a specific antibody protects against septic shock

**DOI:** 10.1038/s41419-019-1808-6

**Published:** 2019-08-05

**Authors:** Chenxi Li, Haiyan Gu, Mingxia Yu, Peng Yang, Meng Zhang, Hongping Ba, Yue Yin, Jing Wang, Bingjiao Yin, Xiaoxi Zhou, Zhuoya Li

**Affiliations:** 1 Department of Immunology, Basic Medicine School, Tongji Medical College, Huazhong University of Science and Technology, Wuhan, Hubei China; 2 Department of Hematology, Tongji Hospital, Tongji Medical College, Huazhong University of Science and Technology, Wuhan, Hubei China

**Keywords:** Target validation, Tumour-necrosis factors, Sepsis, Monocytes and macrophages, Toll-like receptors

## Abstract

Transmembrane TNF-α (tmTNF-α) and secretory TNF-α (sTNF-α) display opposite effects in septic shock. Reducing tmTNF-α shedding can offset the detrimental effects of sTNF-α and increase the beneficial effect of tmTNF-α. We previously developed a monoclonal antibody that is specific for tmTNF-α and does not cross-react with sTNF-α. In this study, we show that this antibody can specifically suppress tmTNF-α shedding by competing with a TNF-α converting enzyme that cleaves the tmTNF-α ectodomain to release sTNF-α. This tmTNF-α antibody significantly inhibited LPS-induced secretion of interleukin (IL)-1β, IL-6, interferon-β, and nitric oxide by monocytes/macrophages, and protected mice from septic shock induced by lipopolysaccharide (LPS) or cecal ligation and puncture, while reducing the bacterial load. The mechanism associated with the protective effect of this tmTNF-α antibody involved promotion of LPS-induced toll-like receptor 4 (TLR4) internalization and degradation by recruiting Triad3A to TLR4. Moreover, the tmTNF-α antibody inhibited LPS-induced activation of nuclear factor-κB and interferon regulatory factor 3 pathways by upregulating expression of A20 and monocyte chemotactic protein-induced protein 1. Similarly, treatment of macrophages with exogenous tmTNF-α suppressed LPS/TLR4 signaling and release of proinflammatory cytokines, indicating that increased levels of tmTNF-α promoted by the antibody contributed to its inhibitory effect. Thus, use of this tmTNF-α antibody for specific suppression of tmTNF-α shedding may be a promising strategy to treat septic shock.

## Introduction

Tumor necrosis factor-α (TNF-α) is first synthesized as transmembrane TNF-α (tmTNF-α), which is then cleaved by the TNF-α converting enzyme (TACE) to release secretory TNF-α (sTNF-α)^1,2^. sTNF-α is widely recognized as a prototypic inflammatory cytokine that plays a pivotal role in the pathogenesis of early endotoxin shock^3–5^. In Gram-negative sepsis, lipopolysaccharide (LPS) binds toll-like receptor 4 (TLR4) and activates NF-κB to produce TNF-α through a MyD88-dependent signaling pathway^6^. sTNF-α induces fever, hypotension, multiple organ dysfunction and death in mice, similar to those evoked by LPS. In addition, sTNF-α promotes neutrophil-mediated tissue injury and amplifies inflammatory cascades by activating macrophages and other types of cells to secrete other proinflammatory cytokines. Although neutralization of TNF-α by monoclonal antibodies can mitigate shock and increase survival in LPS-induced experimental septic shock models^4^, targeted TNF-α therapies have not shown benefits in clinical trials and can even lower patient survival rates by interfering with anti-infection defenses^3,4,7^.

Interestingly, our previous study revealed that, compared with a transient elevation in the levels of serum sTNF-α at 90 min after injection of bacterial into rats, tmTNF-α expression on peritoneal macrophages and liver tissue increased gradually, to peak at 4.5 h after injection, and then declined and stabilized at relatively higher levels up to 24 h after induction of endotoxin shock^8^. This finding indicates a role of tmTNF-α in sepsis. tmTNF-α is a type II transmembrane molecule that binds to TNF receptor (TNFR) to mediate signal transduction to target cells (forward signaling) and itself acts as a receptor that transduces signals in tmTNF-α-bearing cells from inside-to-outside (reverse signaling)^9,10^. In contrast to the pathogenic effects of sTNF-α in sepsis, we and others demonstrated that tmTNF-α functions as an anti-inflammatory factor through forward and reverse signaling. tmTNF-α downregulates LPS- or sTNF-α-induced release of proinflammatory cytokines by reverse signaling in monocytes and macrophages^11,12^. tmTNF-α also inhibits NF-κB activation and decreases IL-6 and MCP-1 production by forward signaling in adipocytes^13^. Moreover, transgenic mice expressing uncleavable tmTNF-α are resistant to LPS and are fully protected from endotoxic shock^14^. These data imply that tmTNF-α, unlike sTNF-α, is beneficial in controlling sepsis and septic shock.

Inhibition of tmTNF-α ectodomain shedding could be a valuable therapeutic strategy to prevent endotoxin shock not only by decreasing release of sTNF-α to attenuate its proinflammatory effects, but also by increasing tmTNF-α expression to enhance its benefits. Indeed, suppression or knockout of TACE, the enzyme that is mainly responsible for tmTNF-α shedding, protects animals from endotoxin shock^8,15,16^. However, TACE has about 76 substrates^17^, and inhibition of TACE may have side effects. We previously developed a tmTNF-α monoclonal antibody (mAb) that specifically recognizes the N-terminal fragment of tmTNF-α and dose not cross-react with sTNF-α^18^. This antibody effectively kills tmTNF-α expressing breast cancer cells^18^ and leukemia cells^19^ by antibody-dependent cell-mediated cytotoxicity and complement-dependent cytotoxicity. In this study, we show that this tmTNF-α mAb can compete with TACE for binding to tmTNF-α and inhibit tmTNF-α ectodomain shedding to protect against endotoxin shock by facilitating LPS-induced TLR4 internalization and degradation, and actively suppressing TLR4 signaling pathways.

## Results

### tmTNF-α antibody specifically inhibits ectodomain shedding of tmTNF-α by competing with TACE for binding to tmTNF-α

**Fig. 1:**
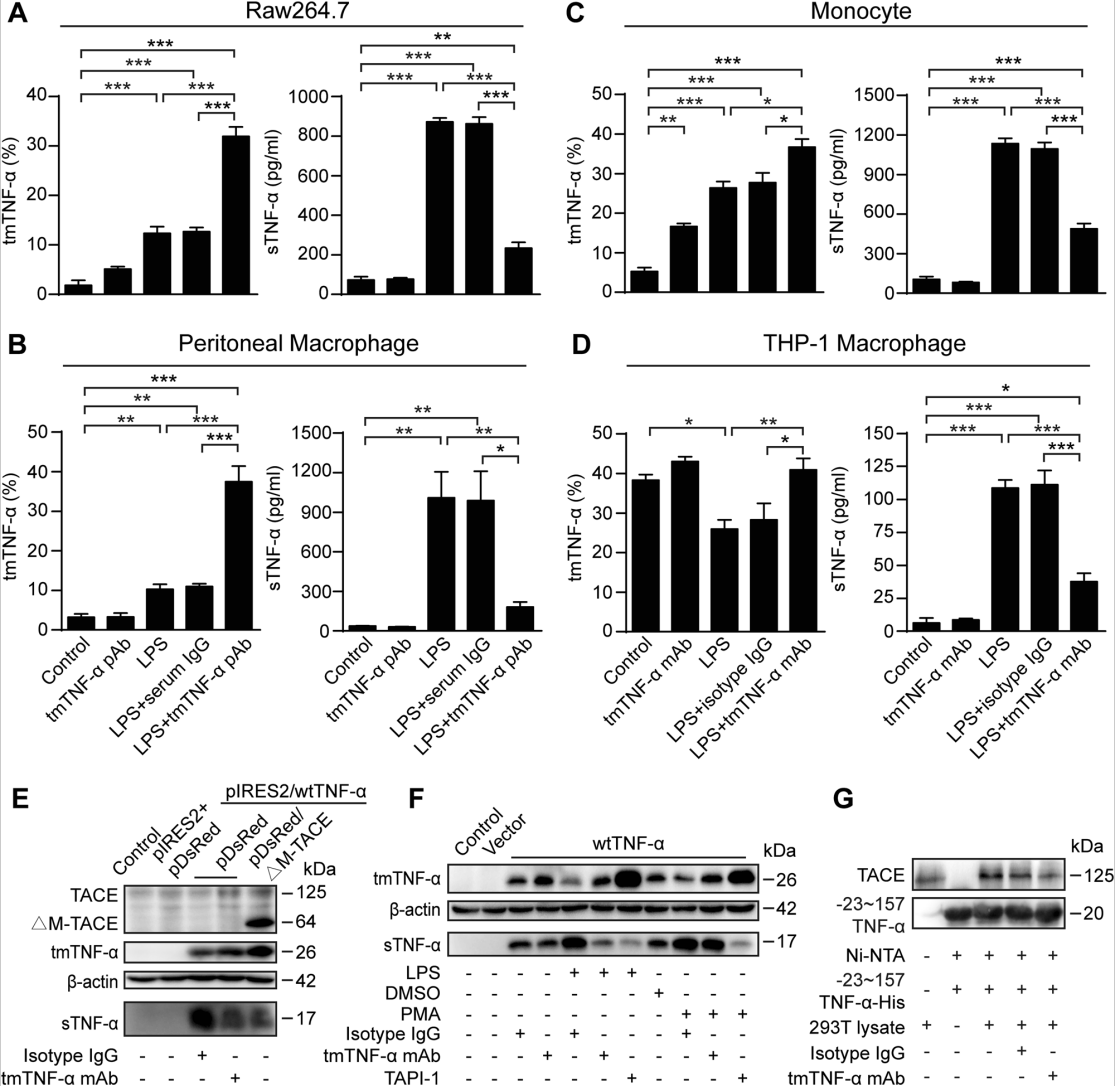
tmTNF-α Ab inhibits the ectodomain shedding of tmTNF-α by blocking TACE binding to tmTNF-α. Raw264.7 (**a**), murine peritoneal macrophages (**b**), peripheral human monocytes (**c**) and THP-1-derived macrophages (**d**) were stimulated with 100 ng/ml LPS, combined with 2 μg/ml murine tmTNF-α polyclonal antibody (pAb) or human tmTNF-α monoclonal Ab (mAB) for 4 h (**a**–**c**) or 1 h (**d**). The same amount of isotype antibody IgG or normal serum IgG served as a control. tmTNF-α on the cell surface was detected by flow cytometry and concentration of sTNF-α in supernatants were determined by ELISA. All quantitative data are presented as means ± SEM of at least three independent experiments. *p < 0.05, **p < 0.01, ***p < 0.001. **e** HEK 293T cells transiently cotransfected for 24 h to express human (h)TNF-α and ▵M-TACE were stimulated with tmTNF-α mAb for 4 h. pIRES2-EGFP (pIRES2) and pDsRed-monomer-N1 (pDsRed) served as empty vector controls. Western blot analysis for the levels of endogenous TACE, ectopically expressed ▵M-TACE and tmTNF-α in total protein and sTNF-α in supernatants. β-actin served as a loading control. **f** HEK 293T cells transiently transfected for 24 h to express hTNF-α were stimulated for 4 h with 100 ng/ml LPS, 60 ng/ml PMA, 2 μg/ml tmTNF-α mAb, or 10 µM TAPI-1. Western blot analysis for expression of 26 kDa tmTNF-α in total protein and 17 kDa sTNF-α in supernatants. **g** The −23~157 TNF-α-His on Ni-NTA-resin was incubated with tmTNF-α mAb or isotype IgG for 4 h, followed by incubation overnight with HEK 293T cell lysates. The resin-bound protein complexes were analyzed by western blotting with antibodies against TACE and TNF-α. Western blot data are representative of three independent experiments

As the epitope recognized by tmTNF-α mAb is closer to the TACE cleavage site, we hypothesized that tmTNF-α Ab may interfere with TACE binding to tmTNF-α and subsequently specifically inhibit tmTNF-α shedding. Since the epitope of human tmTNF-α does no share amino acid sequence homology with murine tmTNF-α, a polyclonal antibody (pAb) was ordered from GL Biochem Ltd (Shanghai, China) using the corresponding epitope-containing peptide conjugated with keyhole limpet hemocyanin. As expected, both human tmTNF-α mAb and murine tmTNF-α pAb significantly increased LPS-induced tmTNF-α expression on the cell surface, but markedly decreased LPS-induced release of sTNF-α in culture supernatants of the murine macrophage cell line Raw264.7, murine peritoneal macrophages, and human monocytes (Fig. 1a–c). The human monocyte cell line THP-1 constitutively expressed high levels of tmTNF-α (Supplementary Fig. 1A). Phorbol myristate acetate (PMA), used to differentiate THP-1 cells, is also an activator of TACE^20^ and induced tmTNF-α shedding to decrease tmTNF-α expression levels and increase sTNF-α release (Supplementary Fig. 1B, C). However, THP-1-derived macrophages still expressed a relatively high level of tmTNF-α in the absence of LPS stimulation. Therefore, LPS-mediated tmTNF-α shedding induced lower levels of tmTNF-α compared with the control. Similarly, tmTNF-α mAb significantly blocked tmTNF-α shedding after LPS stimulation (Fig. 1d). These data indicate a possible role for antibodies in suppression of tmTNF-α processing.

To further verify the function of tmTNF-α Ab in tmTNF-α shedding, we transiently transfected HEK 293T cells with TNF-α. Western blotting showed that ectopically expressed 26 kDa tmTNF-α was rapidly cleaved into 17 kDa sTNF-α, whereas coexpression of TNF-α and ▵M-TACE, a dominant negative mutant that lacks the metalloprotease domain, significantly blocked tmTNF-α shedding, indicating a basic activation of endogenous TACE in HEK 293T cells (Fig. 1e). Importantly, tmTNF-α mAb not only inhibited tmTNF-α processing, but also suppressed both LPS- and PMA-induced tmTNF-α shedding, although the inhibitory effect of TAPI-1, a TACE inhibitor, was stronger (Fig. 1f). Pulldown results showed that TACE binding to tmTNF-α was evidently blocked by tmTNF-α mAb (Fig. 1g), suggesting that tmTNF-α Ab and TACE compete for binding to tmTNF-α.

As TNFR is a TACE substrate, a TACE inhibitor suppresses not only tmTNF-α cleavage but also release of soluble TNFR (sTNFR), which can buffer the effects of sTNF-α. Indeed, TAPI-1 inhibited LPS-induced sTNFR1 release into THP-1 supernatants, yet tmTNF-α mAb had no effect (Supplementary Fig. 1D). This outcome was confirmed in a bioassay, in which Cas9-CRISPR was used to silence TNF-α expression in THP-1 cells (Supplementary Fig. 1E). Supernatants containing sTNFR obtained from LPS-stimulated TNF-α-KO THP-1 cells significantly blocked sTNF-α-mediated cytotoxicity. The suppressive effect of LPS was not affected by supernatants from cells cotreated with tmTNF-α mAb, but was reversed by supernatants from cells cotreated with TAPI-1 (Supplementary Fig. 1F). These data suggest that tmTNF-α mAb, unlike the TACE inhibitor, does not affect LPS-induced release of sTNFR and its buffering capacity.

### Increasing tmTNF-α expression by tmTNF-α Ab suppresses LPS response of monocytes/macrophages and TNFR2 mediates the inhibitory effects of tmTNF-α

**Fig. 2:**
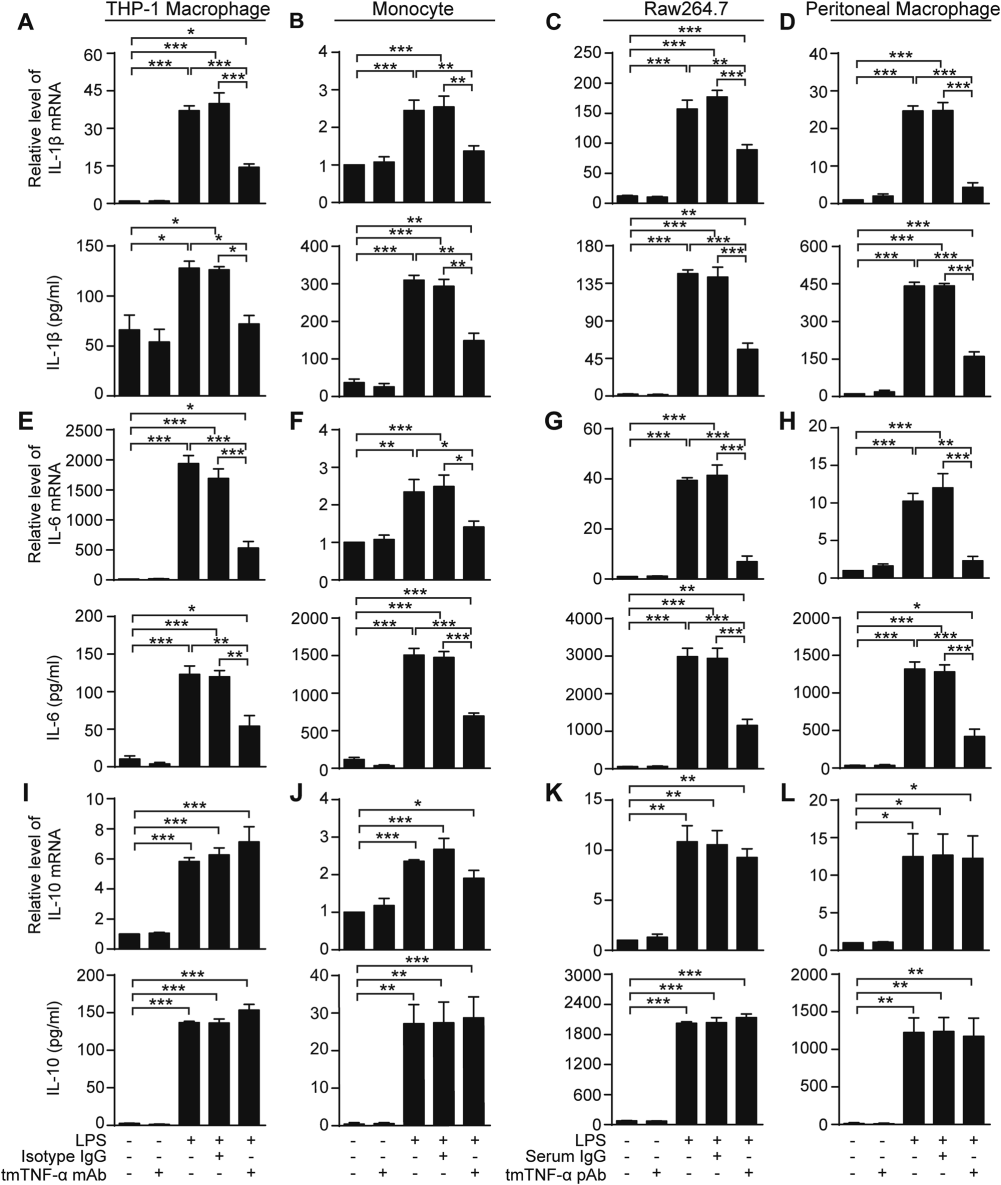
tmTNF-α Ab decreases LPS-induced production of proinflammatory cytokines. THP-1-derived macrophages (**a**, **e**, **i**), primary human monocytes (**b**, **f**, **j**), Raw264.7 (**c**, **g**, **k**), and murine peritoneal macrophages (**d**, **h**, **l**) were stimulated with 100 ng/ml LPS, combined with 2 μg/ml human tmTNF-α mAb or murine tmTNF-α pAb. The same amount of isotype antibody IgG or normal serum IgG served as a control. The stimulation time was 4 h for detection of mRNA levels by real-time PCR and 10 h for detection of cytokine concentrations by ELISA. The production of IL-1β (**a**–**d**), IL-6 (**e**–**h**), and IL-10 (**i**–**l**) was detected at mRNA (upper panels) and protein (lower panels) levels. All data are presented as means ± SEM of at least three independent experiments. *p < 0.05, **p < 0.01, ***p < 0.001

Next, we tested the impact of tmTNF-α Ab on LPS-induced production of pro- and anti-inflammatory cytokines. Both tmTNF-α mAb and pAb significantly inhibited LPS-induced production of proinflammatory cytokines IL-1β (Fig. 2a–d) and IL-6 (Fig. 2e–h) at both the mRNA and protein level in murine and human macrophages and monocytes. However, tmTNF-α Abs had no effect on LPS-induced production of anti-inflammatory cytokine IL-10 (Fig. 2i–l). These data indicate that tmTNF-α Abs induce LPS resistance in monocytes/macrophages.

**Fig. 3:**
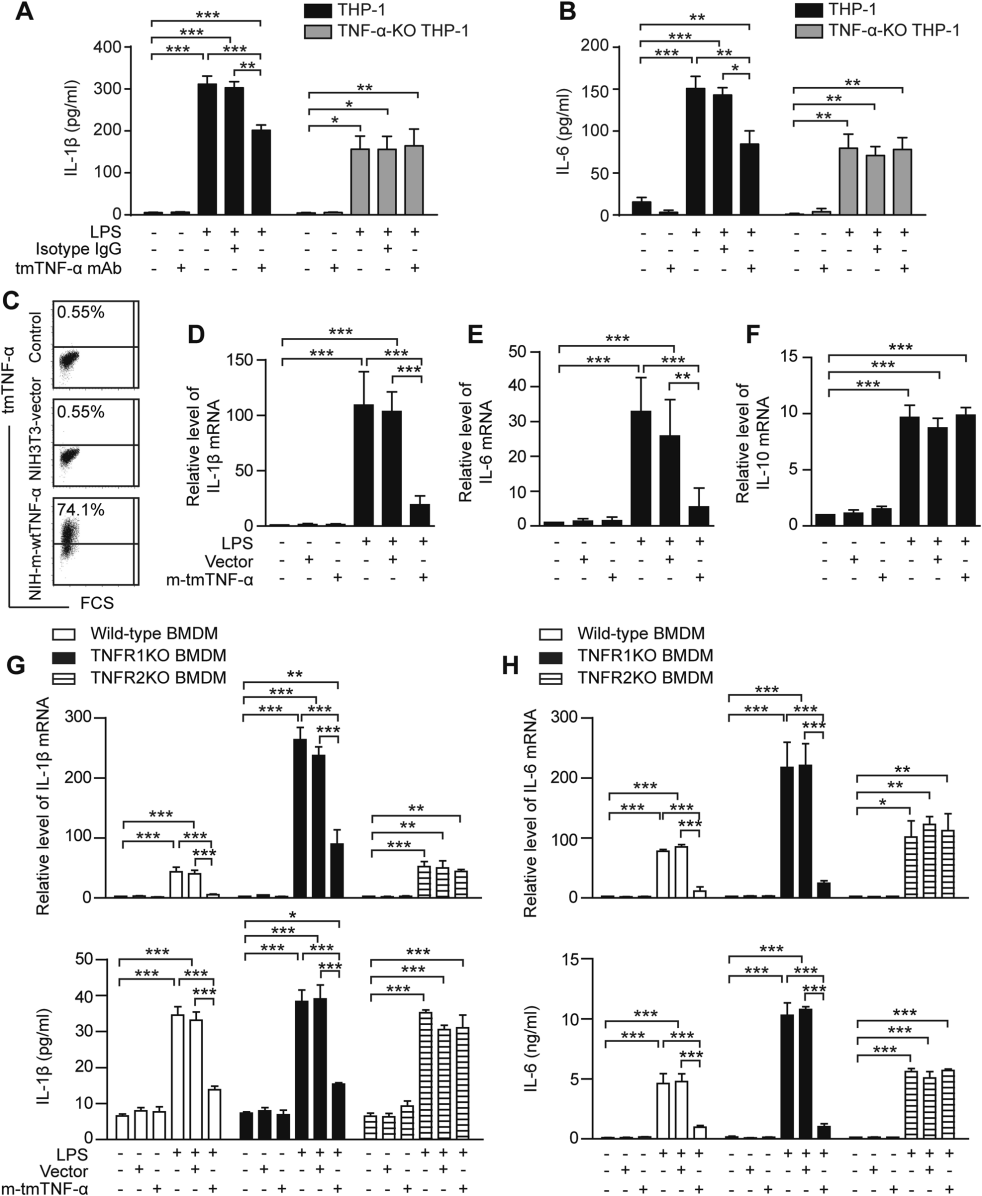
Exogenous tmTNF-α decreases LPS-induced production of proinflammatory cytokines. **a**, **b** tmTNF-α-expressing or TNF-α-KO THP-1 cells were stimulated with 100 ng/ml LPS, combined with 2 μg/ml tmTNF-α mAb or isotype IgG for 12 h. The concentrations of IL-1β and IL-6 in supernatants were determined by ELISA. **c** NIH3T3 cells stably transfected with murine TNF-α. Ectopic expression of tmTNF-α on the cell surface was detected by flow cytometry. tmTNF-α on 4% paraformaldehyde-fixed NIH3T3-wtTNF-α cells was cocultured with Raw264.7 (**d**–**f**) or BMDM (**g**, **h**) from wild type, TNFR1KO, or TNFR2KO mice at an effector/target ratio of 10:1 for 30 min, followed by 100 ng/ml LPS stimulation. The empty vector transfected NIH3T3 cells served as a control. The production of IL-1β (**d**, **g**), IL-6 (**e**, **h**) and IL-10 (**f**) was detected at mRNA (4 h after stimulation) and protein (10 h after stimulation) levels by real-time PCR and ELISA, respectively. All data are presented as means ± SEM of at least three independent experiments. *p < 0.05, **p < 0.01, ***p < 0.001

To test, whether the effect of tmTNF-α Ab is mediated through its action on TNF-α, we added the antibody to tmTNF-α-expressing or TNF-α-KO THP-1 cells stimulated with LPS for 12 h. We found that LPS induced release of IL-1β and IL-6 in both tmTNF-α-expressing and TNF-α-KO THP-1 cells, although IL-1β and IL-6 levels were decreased by TNF-α-KO. However, tmTNF mAb significantly suppressed LPS-induced production of these cytokines in tmTNF-α-expressing, but not in TNF-α-KO THP-1 cells (Fig. 3a, b), indicating that tmTNF mAb had no direct effect on the LPS response itself, but instead induced LPS resistance through its action on TNF-α. As demonstrated above, tmTNF Abs increased tmTNF-α expression and decreased sTNF-α release by inhibition of tmTNF-α shedding. Although decreasing sTNF-α release attenuated its proinflammatory effects, increasing tmTNF-α levels induced by tmTNF-α Abs might contribute to the induction of LPS resistance. To test this possibility, we cocultured 4% paraformaldehyde-fixed NIH3T3 cells overexpressing murine tmTNF-α (Fig. 3c) with Raw264.7 cells and bone marrow-derived macrophages (BMDM) from wild-type, TNFR1KO, and TNFR2KO mice. Similar to the effect of tmTNF-α Abs, direct addition of exogenous tmTNF-α to Raw264.7 cells significantly inhibited LPS-induced mRNA transcription of proinflammatory cytokines (Fig. 3d, e), but did not affect IL-10 transcription (Fig. 3f) in Raw264.7, indicating that tmTNF-α actively induces LPS resistance in macrophages. In addition, the inhibitory effect of tmTNF-α on LPS-induced production of IL-1β (Fig. 3g) and IL-6 (Fig. 3h) could be totally blocked by TNFR2KO, but not by TNFR1KO in BMDM, although mRNA levels of these proinflammatory cytokines and IL-6 secretion were higher in TNFR1KO BMDM than those in wild-type and TNFR2KO BMDM.

### tmTNF-α Ab protects against LPS- and CLP-induced septic shock

**Fig. 4:**
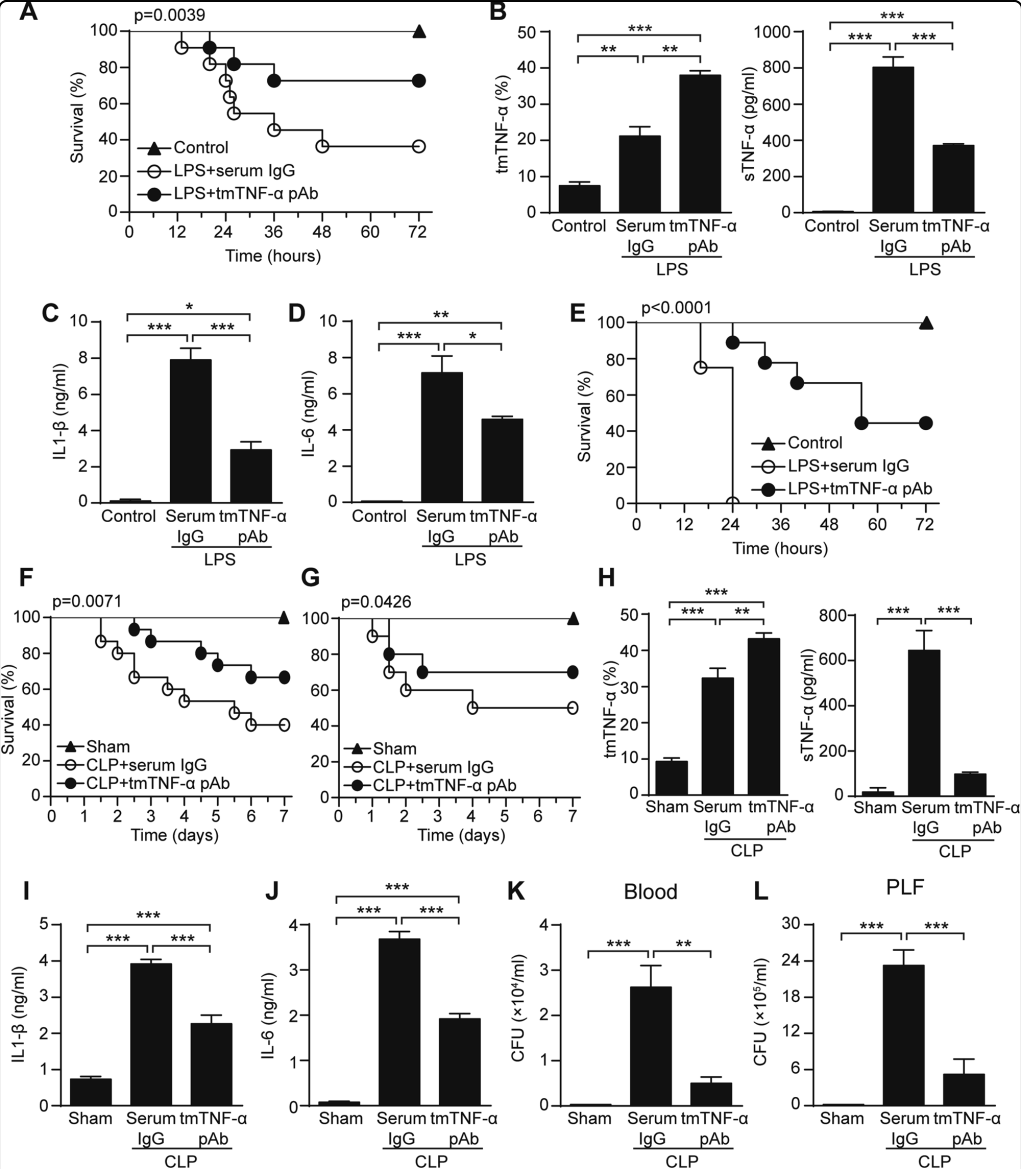
tmTNF-α Ab protects against LPS/CLP-induced septic shock. C57BL/6 mice were treated i.p. with 600 μg tmTNF-α pAb or normal serum IgG for 30 min prior to intraperitoneal injection of 30 mg/kg LPS. **a** Kaplan–Meier survival curves of mice (*n* = 11, each group). **b** tmTNF-α expression on the cell surface of peritoneal macrophages or serum concentrations of sTNF-α detected by flow cytometry or ELISA, respectively, at 4 h after LPS stimulation. **c**, **d** Serum levels of IL-1β and IL-6 were measured by ELISA at 6 h after LPS treatment. **e** Kaplan–Meier survival curves of mice challenged with intraperitoneal injection of 50 mg/kg LPS after pretreatment with 600 μg tmTNF-α pAb or normal serum IgG for 30 min (*n* = 9, each group). C57BL/6 mice were intraperitoneally injected with 600 μg tmTNF-α pAb or normal serum IgG immediately (**f**) or at 5 h (**g**) after the CLP operation. Kaplan–Meier survival curves of mice (*n* = 15 for **f**, *n* = 10 for **g**, each group). tmTNF-α expression on the cell surface of peritoneal macrophages and serum concentrations of sTNF-α (**h**), IL-1β (**i**) and IL-6 (**j**) were determined by flow cytometry and ELISA, respectively. The bacterial load in peripheral blood (**k**) and peritoneal lavage fluid (**l**) at 24 h after the CLP operation. *p < 0.05, **p < 0.01, ***p < 0.001

To test whether tmTNF-α Ab can prevent endotoxin shock, mice were intraperitoneally injected with LPS (30 mg/kg). A 30 min-pretreatment with tmTNF-α pAb significantly increased animal survival (from 36 to 73%) (Fig. 4a), which was accompanied by increased tmTNF-α expression in peritoneal macrophages and decreased serum levels of sTNF-α (Fig. 4b), IL-1β (Fig. 4c) and IL-6 (Fig. 4d) after LPS challenge. In addition, in mice injected with a lethal dose of LPS (50 mg/kg), tmTNF-α pAb still markedly increased animal survival (from 0 to 44%) and protected against endotoxin shock (Fig. 4e).

We used another septic shock animal model, cecal ligation and puncture (CLP), in which sepsis is induced by a polymicrobial infection in the abdominal cavity that involves translocation of bacteria and toxins into the bloodstream, to confirm the protective activity of the tmTNF-α antibody. Treatment of mice with tmTNF-α pAb immediately or 5 h after the CLP operation also evidently increased survival from 40% to 66.7% and from 50% to 70% (Fig. 4f, g), respectively. Similarly, the antibody inhibited tmTNF-α shedding (Fig. 4h) and decreased IL-1β and IL-6 plasma levels (Fig. 4i, j), indicating again that tmTNF-α pAb protected against septic shock. Since complete neutralization of TNF-α aggravates infection by interfering with defense mechanisms^4^, we evaluated whether tmTNF-α Ab affected host antibacterial defenses. Interestingly, tmTNF-α pAb markedly reduced bacterial load in blood and peritoneal lavage fluid (PLF) at 24 h after the CLP operation (Fig. 4k, l), reserving the anti-infection effect of TNF-α.

### tmTNF-α Ab facilitates LPS-induced TLR4 internalization and degradation

**Fig. 5:**
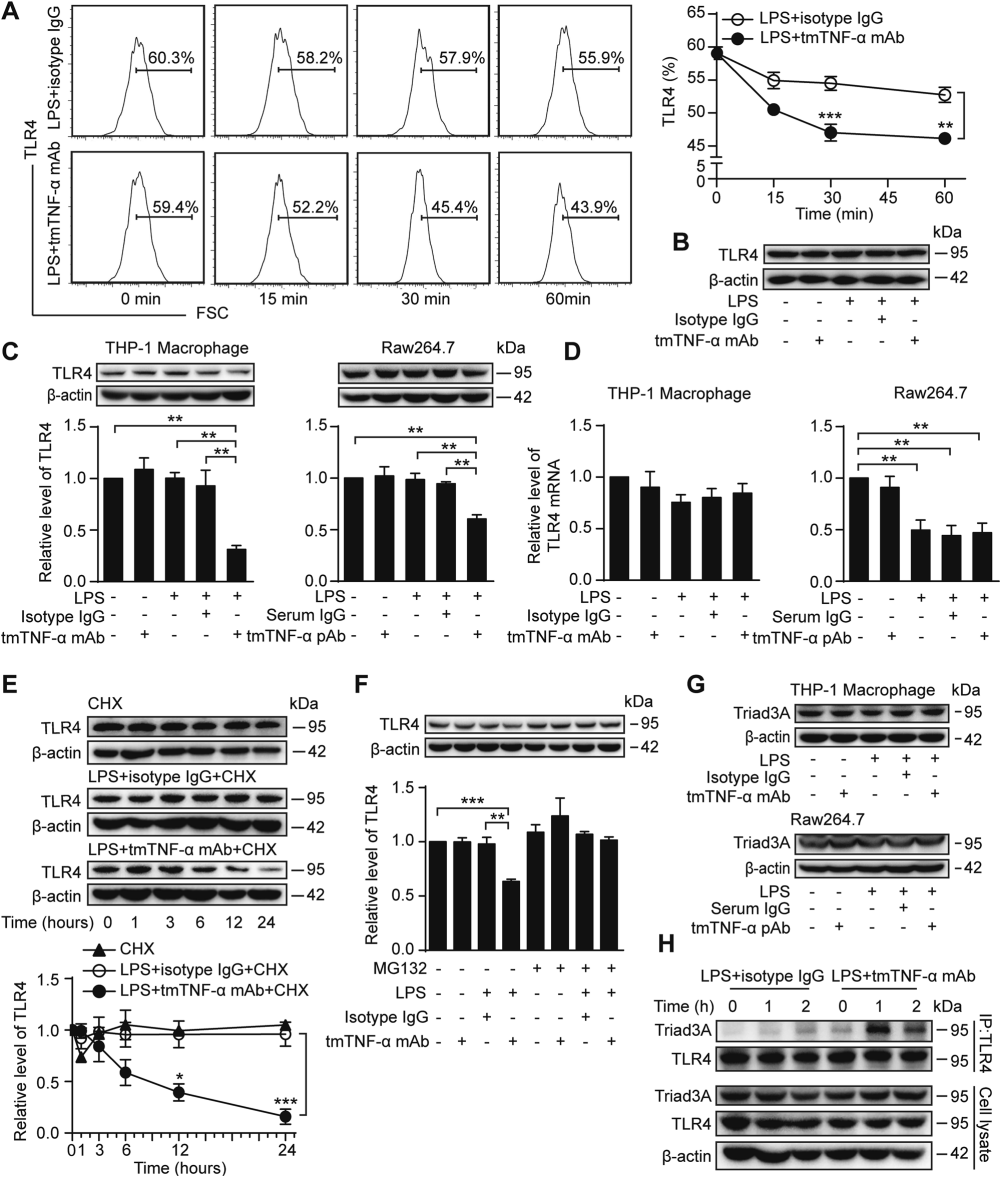
tmTNF-α Ab facilitates LPS-induced TLR4 internalization and degradation. THP-1-derived macrophages were stimulated with 100 ng/ml LPS and 2 μg/ml tmTNF-α mAb or isotype IgG for indicated time points. **a** TLR4 expression on the cell surface was evaluated by flow cytometry. Representative images of FCM on the left, and quantitative data on the right. **b** Western blot analysis of TLR4 expression after stimulation for 1 h. THP-1-derived and Raw264.7 macrophages were stimulated with 100 ng/ml LPS, combined with 2 μg/ml tmTNF-α mAb or tmTNF-α pAb, respectively. Isotype antibody IgG or normal serum IgG served as a control. **c** Representative images of western blot analysis of TLR4 expression at 12 h after stimulation (upper) and their quantitative data (lower). **d** Relative levels of TLR4 mRNA were assessed by real-time PCR 4 h after stimulation. **e** THP-1-derived macrophages were stimulated with LPS and tmTNF-α mAb for indicated time points in the presence of 10 μg/ml cycloheximide. Representative images of western blot analysis of TLR4 expression (upper) and their quantitative data (lower). **f** THP-1-derived macrophages were treated for 4 h with 10 μM MG132 prior to the stimulation with LPS and tmTNF-α mAb for 12 h. Representative images of western blot analysis of TLR4 expression (upper) and their quantitative data (lower). **g** Western blot analysis of Triad3A expression in THP-1-derived or Raw264.7 macrophages stimulated with LPS and tmTNF-α mAb or pAb for 12 h, respectively. **h** Representative images of IP/western blot analysis of Triad3A recruited to TLR4 in THP-1-derived macrophages stimulated with LPS and tmTNF-α mAb for indicated time points. All quantitative data are presented as means ± SEM of at least three independent experiments. *p < 0.05, **p < 0.01, ***p < 0.001

Since LPS induces internalization of TLR4^21^, we tested cell surface levels of TLR4 by flow cytometry. In THP-1-derived macrophages, tmTNF-α mAb markedly promoted a LPS-induced decline in TLR4 levels on the cell surface within 60 min of stimulation (Fig. 5a) but did not affect TLR production at 1 h after stimulation (Fig. 5b), indicating that tmTNF-α mAb had an enhancing effect on LPS-induced TLR4 internalization. However, both tmTNF-α mAb and pAb evidently decreased total TLR4 expression at 12 h after LPS stimulation in THP-1-derived and Raw264.7 macrophages, respectively, but LPS or tmTNF-α Abs alone had no effect (Fig. 5c). We next used real-time PCR to test whether tmTNF-α Ab inhibited TLR4 production. Neither LPS nor tmTNF-α mAb affected TLR4 mRNA transcription in THP-1-derived macrophages, but LPS suppressed TLR4 transcription in Raw264.7 macrophages, which was not affected by tmTNF-α pAb (Fig. 5d). These results suggested that tmTNF-α Ab had no effect on TLR4 gene expression. Instead, the tmTNF-α Ab might affect TLR4 levels by promoting TLR4 degradation. Indeed, tmTNF-α mAb did induce TLR4 degradation in the presence of LPS, although LPS itself did not induce TLR4 degradation after a 24 h treatment of THP-1-derived macrophages with cycloheximide (Fig. 5e). This effect was completely blocked by the proteasome inhibitor MG132 at 12 h after LPS stimulation (Fig. 5f). Triad3A is known to interact with TLR4, promoting its ubiquitination and degradation^22,23^. Although neither LPS nor tmTNF-α Abs affected Triad3A expression in THP-1-derived and Raw 264.7 macrophages (Fig. 5g), IP/western blot analysis revealed that LPS induced trace amounts of Triad3A to be recruited to TLR4, whereas tmTNF-α mAb promoted the recruitment of substantial amounts of Triad3A to TLR4 at 1 h and 2 h after LPS stimulation (Fig. 5h), indicating that this antibody facilitated Triad3A-dependent TLR4 degradation. These data suggested that tmTNF-α Ab promotes LPS-induced TLR4 internalization at an early stage and degradation at a later stage to induce LPS resistance.

As tmTNF-α Ab increased the tmTNF-α expression levels, we tested the ability of tmTNF-α to promote TLR4 internalization and degradation. Because THP-1 is a human cell line, we stably transfected HEK 293T cells with full-length of human TNF-α and confirmed the high level of tmTNF-α expression on the cell surface (>75%, Supplementary Fig. 2A). Exogenous human tmTNF-α on fixed 293T cells or murine TNF-α on fixed NIH 3T3 cells was added to THP-1-derived or Raw 264.7 macrophages, respectively, at a ratio of 10:1. As expected, tmTNF-α significantly increased LPS-induced TLR4 internalization within 60 min (Supplementary Fig. 2B) and also induced TLR4 degradation 12 h after LPS stimulation (Supplementary Fig. 2C, D), suggesting that increased amounts of tmTNF-α mediated the effect of the Ab on TLR4.

### tmTNF-α Ab inhibits the MyD88-dependent TLR4 signaling pathway

**Fig. 6:**
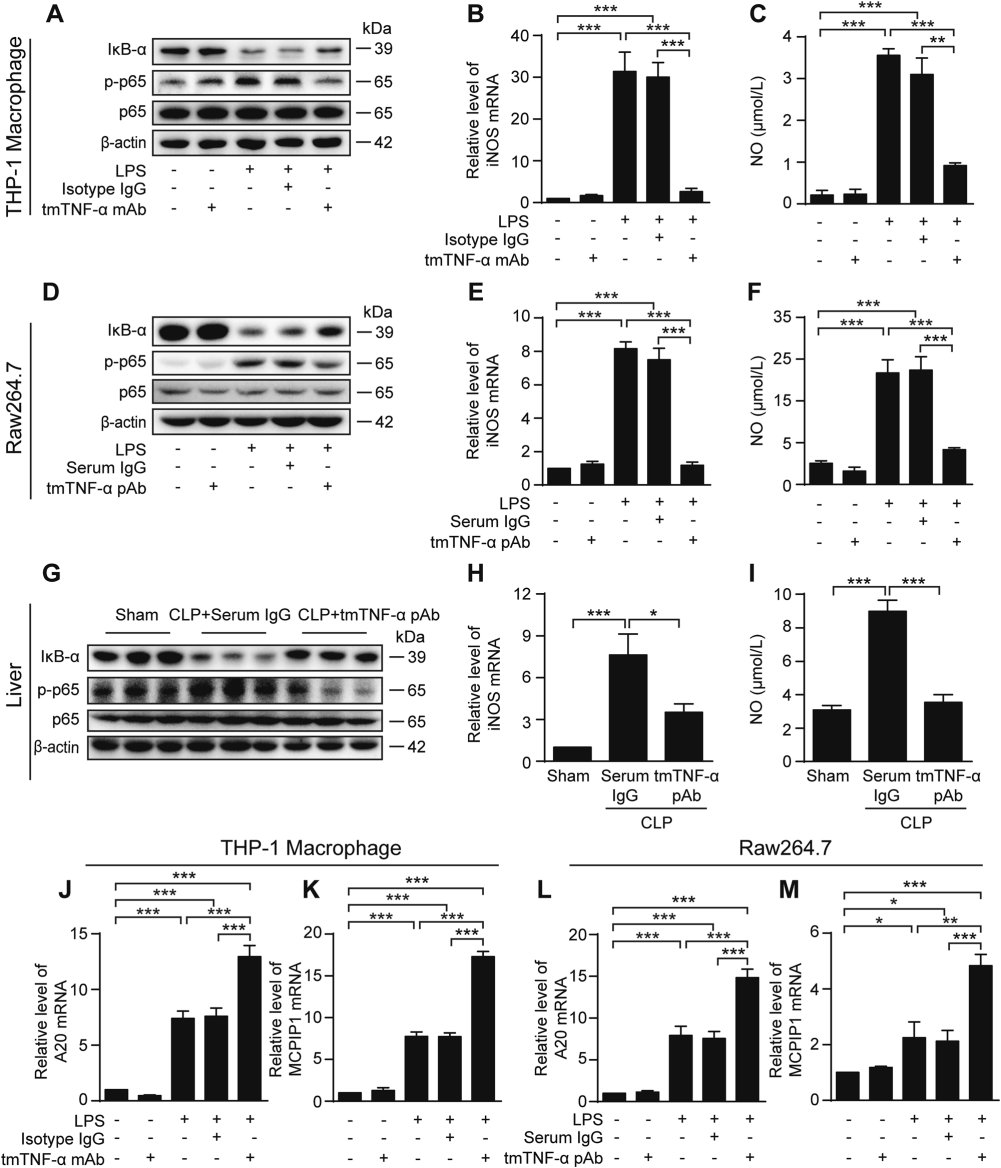
tmTNF-α Ab inhibits the MyD88-dependent TLR4 signaling pathway. THP-1-derived or Raw264.7 macrophages were stimulated with 100 ng/ml LPS, combined with 2 μg/ml tmTNF-α mAb or pAb, respectively. Isotype antibody IgG or normal serum IgG served as a control. **a**, **d** Representative western blot of three independent experiments for IκBα degradation and p65 phosphorylation in THP-1-derived (1 h after stimulation) or Raw264.7 macrophages (45 min after stimulation). Relative levels of iNOS mRNA were assessed by real-time PCR at 4 h (**b**, **e**), and NO production at 10 h after stimulation (**c**, **f**). All quantitative data are presented as means ± SEM of at least three independent experiments. Mice were intraperitoneally injected with 600 μg tmTNF-α pAb or normal serum IgG immediately after the CLP operation (*n* = 6 each group). Western blot analysis of IκBα degradation and p65 phosphorylation (**g**) and relative levels of iNOS mRNA in the liver (**h**), and plasma levels of NO (**i**) 24 h after the CLP operation. THP-1-derived or Raw264.7 macrophages were stimulated for 4 h with LPS and tmTNF-α mAb or pAb, respectively. Relative mRNA levels of A20 (**j**, **l**) and MCPIP1 (**k**, **m**) were assessed by real-time PCR. All quantitative data are presented as means ± SEM of at least three independent experiments. *p < 0.05, **p < 0.01, ***p < 0.001

Since LPS/TLR4 activates the MyD88-dependent NF-κB pathway that mediates production of proinflammatory cytokines^6^, we investigated the effect of tmTNF-α Ab on this pathway. tmTNF-α mAb and pAb remarkably inhibited LPS-induced IκBα degradation and p65 phosphorylation in THP-1-derived (Fig. 6a) and Raw264.7 macrophages (Fig. 6d), respectively. As a result, tmTNF-α Abs suppressed LPS-induced mRNA transcription of the NF-κB targeting gene iNOS (Fig. 6b, e) and NO release (Fig. 6c, f) in these macrophages and in human monocytes (Supplementary Fig. 3A, B). A similar phenomenon was observed in vivo in CLP-induced septic shock, wherein tmTNF-α pAb effectively suppressed IκBα degradation and p65 phosphorylation (Fig. 6g) and also reduced levels of of iNOS mRNA in liver tissues and NO in plasma (Fig. 6h, i). In addition, tmTNF-α Abs also inhibited LPS-induced phosphorylation of ERK, JNK, and p38 in vitro (Supplementary Fig. 3C, D) and in vivo (Supplementary Fig. 3E). These phenomena were observed upon adding exogenous tmTNF-α to Raw264.7 and BMDM (Supplementary Fig. 3F–I), but were totally blocked in TNFR2KO cells (Supplementary Fig. 3G, I), suggesting that increases in tmTNF-α following antibody treatment indeed suppressed LPS/TLR4 signaling.

To explore the mechanism of the suppressive effect of tmTNF-α Ab on LPS-induced NF-κB activation, we assessed levels of negative regulators of NF-κB by real-time PCR. tmTNF-α mAb and pAb significantly increased LPS-induced mRNA transcription of A20 and monocyte chemotactic protein-induced protein 1 (MCPIP1) in THP-1-derived (Fig. 6j, k) and Raw264.7 macrophages (Fig. 6l, m), respectively. Exogenous tmTNF-α also consistently upregulated mRNA transcription of these two molecules (Supplementary Fig. 4A, B). However, tmTNF-α Abs did not markedly affect LPS-induced mRNA transcription of interleukin 1 receptor associated kinase 3 (IRAK)-M (Supplementary Fig. 4C, G), src homology 2 domain–containing inositol-5-phosphatase 1 (SHIP1, Supplementary Fig. 4D, H), suppressor of cytokine signaling (SOCS) 1 (Supplementary Fig. 4E, I) and SOCS3 (Supplementary Fig. 4F, J). These findings indicate that increases in tmTNF-α promoted by the tmTNF-α Ab negatively regulated the MyD88-dependent TLR4 signaling pathway upon LPS stimulation.

### tmTNF-α Ab suppresses TRIF-dependent TLR4 signaling pathway

**Fig. 7:**
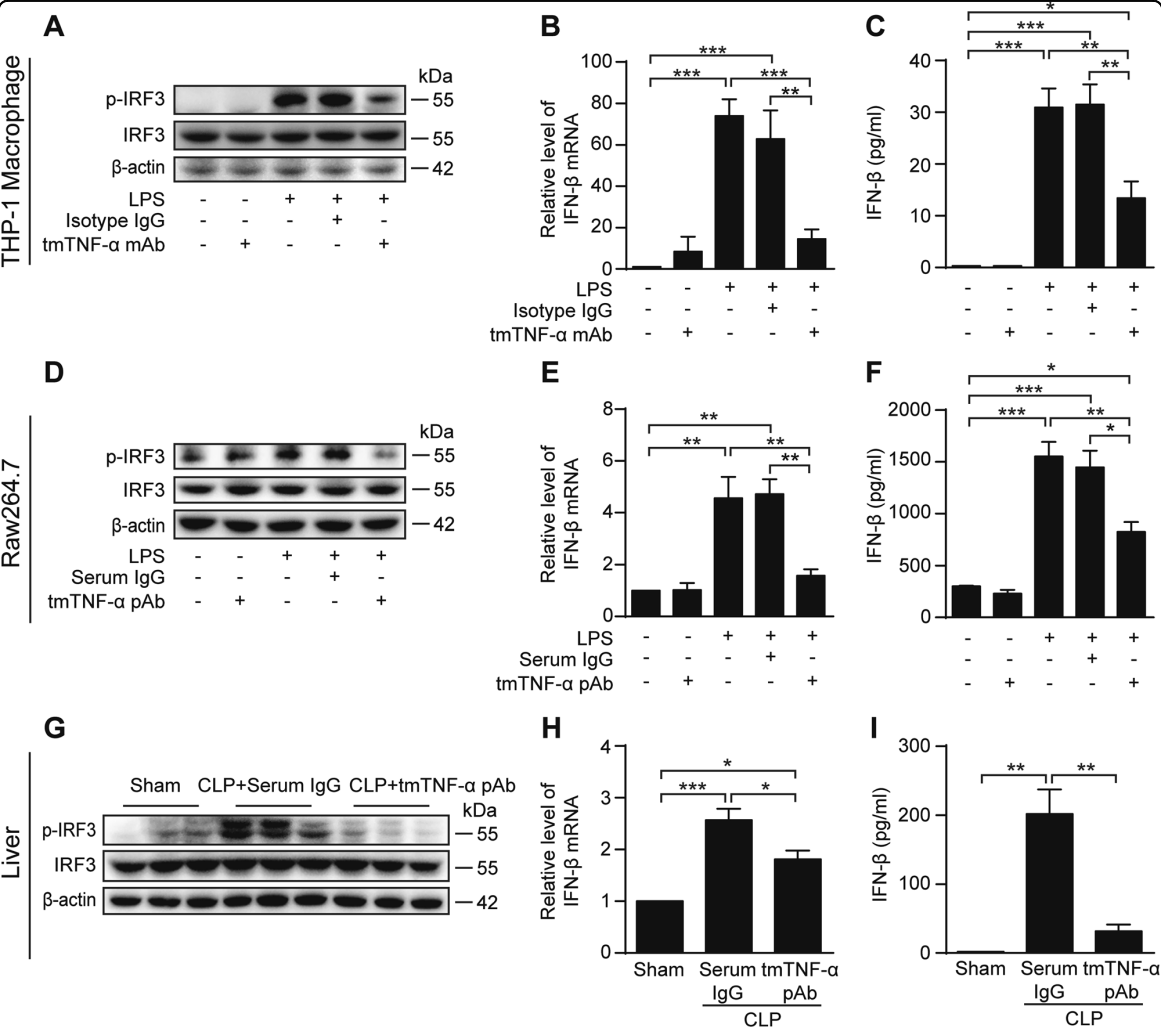
tmTNF-α Ab inhibits the TRIF-dependent TLR4 signaling pathway. THP-1-derived or Raw264.7 macrophages were stimulated with 100 ng/ml LPS, combined with 2 μg/ml tmTNF-α mAb or pAb, respectively. Isotype antibody IgG or normal serum IgG served as a control. **a**, **d** Representative western blot analysis of three independent experiments for IRF3 phosphorylation in THP-1-derived (1 h after stimulation) or Raw264.7 macrophages (45 min after stimulation). Relative levels of IFN-β mRNA were assessed by real-time PCR at 4 h (**b**, **e**), and IFN-β release was detected by ELISA at 10 h (**c**, **f**) after stimulation. All quantitative data are presented as means ± SEM of at least three independent experiments. Mice were intraperitoneally injected with 600 μg tmTNF-α pAb or normal serum IgG immediately after the CLP operation (*n* = 6 each group). Western blot analysis of IRF3 phosphorylation (**g**) and relative levels of IFN-β mRNA in the liver (**h**) and plasma levels of IFN-β (**i**) at 24 h after the CLP operation. *p < 0.05, **p < 0.01, ***p < 0.001

We then evaluated the effect of tmTNF-α Ab on another LPS/TLR signaling pathway, the TRIF-dependent signaling pathway. tmTNF-α mAb and pAb significantly blocked LPS-induced phosphorylation of interferon regulatory factor 3 (IRF3) in THP-1-derived and Raw264.7 macrophages (Fig. 7a, d), respectively. Consequently, the antibodies inhibited LPS-induced mRNA transcription (Fig. 7b, e) and protein production (Fig. 7c, f) from the IRF3-targeting gene IFN-β in THP-1-derived and Raw264.7 macrophages and in human monocytes (Supplementary Fig. 5A, B). Moreover, tmTNF-α pAb evidently reduced IRF3 phosphorylation (Fig. 7g) and IFN-β transcription (Fig. 7h) in the liver and serum levels of IFN-β (Fig. 7i) 24 h after the CLP operation. However, tmTNF-α Abs did not markedly affect LPS-induced mRNA transcription of negative regulators including deubiquitinating enzyme A (DUBA, Supplementary Fig. 5C, F), peptidyl-prolyl *cis*/*trans* isomerase NIMA-interacting 1 (Pin1, Supplementary Fig. 5D, G) and Src homology 2 domain-containing protein tyrosine phosphatase-2 (SHP2, Supplementary Fig. 5E, H) in THP-1-derived and Raw264.7 macrophages. Our in vitro and in vivo results indicated that Ab-dependent increases in tmTNF-α negatively regulate LPS-stimulation of the TRIF-dependent TLR4 signaling pathway.

## Discussion

Here we demonstrated that tmTNF-α Ab inhibited tmTNF-α shedding by competing with TACE for binding to tmTNF-α. This antibody induced LPS resistance of monocytes/macrophages and protected mice against LPS- and CLP-induced septic shock by suppressing TLR4 signaling pathways.

Previously, we developed a tmTNF-α antibody that effectively kills tmTNF-α expressing tumor cells in vitro and in vivo^18,19^. In this study, we explored the novel function of this antibody, which involves specific blockage of ectodomain shedding of tmTNF-α by competing with TACE for the substrate binding. This antibody selectively inhibited sTNF-α release to reduce its detrimental effects while increasing tmTNF-α expression to exert its beneficial effect of inducing LPS resistance in vitro and in vivo. In addition, in contrast to the TACE inhibitor, the tmTNF-α Ab did not affect LPS-induced, TACE-dependent TNFR1 shedding. This is helpful for neutralization of sTNF-α that was reduced by the antibody, thus further alleviating its detrimental effects. Moreover, binding of released sTNFR1 to tmTNF-α initiates reverse signaling, which extends the protective function of tmTNF-α from local to distant through conversion of its action from juxtacrine to retrocrine. This is beneficial for controlling sepsis and septic shock.

Our results revealed that the antibody induced LPS resistance of monocytes/macrophages in vitro, showing downregulated LPS-induced production of NO, IL-1β, IL-6 and IFN-β, and conferred protection against LPS and CLP-induced septic shock, manifested as reduced inflammation and increased survival. The benefit of the antibody can mainly be attributed to enhanced tmTNF-α-mediated anti-inflammatory activity, which was further confirmed by our results showing that exogenous tmTNF-α actively suppressed LPS-induced production of proinflammatory cytokines through TNFR2 in macrophages. Increasing evidence indicates that tmTNF-α exerts protective effect against bacterial infections, chronic inflammation, and autoimmunity diseases^24^. As a ligand, tmTNF-α attenuates the inflammatory processes caused by mycobacterial pleurisy in association with TNFR2 expression on myeloid cells^25^. Interactions between tmTNF-α and TNFR2 are important for the expansion and function of Treg cells^26^ and for activation of myeloid-derived suppressive cells to exert immune suppressive activities^27^. On the other hand, as a receptor, tmTNF-α induces LPS resistance to suppress proinflammatory cytokine production through MAPK/ERK and NF-κB pathways^13,28^, to control inflammation by induction of TGF-β expression through reverse signaling^29^.

Our data revealed that the Ab-mediated increases in tmTNF-α expression reduced the response of macrophages to LPS at the receptor and postreceptor levels. Upon LPS stimulation, the MyD88-dependent signaling pathway is first initiated at the plasma membrane and subsequently activates NF-κB to drive production of proinflammatory mediators. Then, a TRIF-dependent signaling pathway is triggered after TLR internalization to activate IRF3 and induce type I interferon production. Eventually, TLR4 is ubiquitinated and degraded^21^. We found that increased tmTNF-α expression levels promoted by tmTNF-α Ab facilitated earlier LPS-induced TLR4 internalization and later TLR4 degradation. These phenomena were also observed upon addition of exogenous tmTNF-α to THP-1-derived macrophages exposed to LPS, indicating that tmTNF-α mediated the inhibitory action of the Ab toward TLR4. Notably, LPS alone neither induced TLR4 expression nor affected its degradation in THP-1-derived macrophages. The former result is in line with a report by Aerbajinai et al.^30^ Furthermore, tmTNF-α Ab induced recruitment of the E3 ubiquitin-protein ligase Triad3A to TLR4 to promote TLR4 degradation through the ubiquitin–proteasome-dependent pathway, as tmTNF-α Ab-induced TLR4 degradation could be completely blocked by a proteasome inhibitor. Our data thus revealed a novel mechanism for tmTNF-α-induced LPS resistance.

**Fig. 8:**
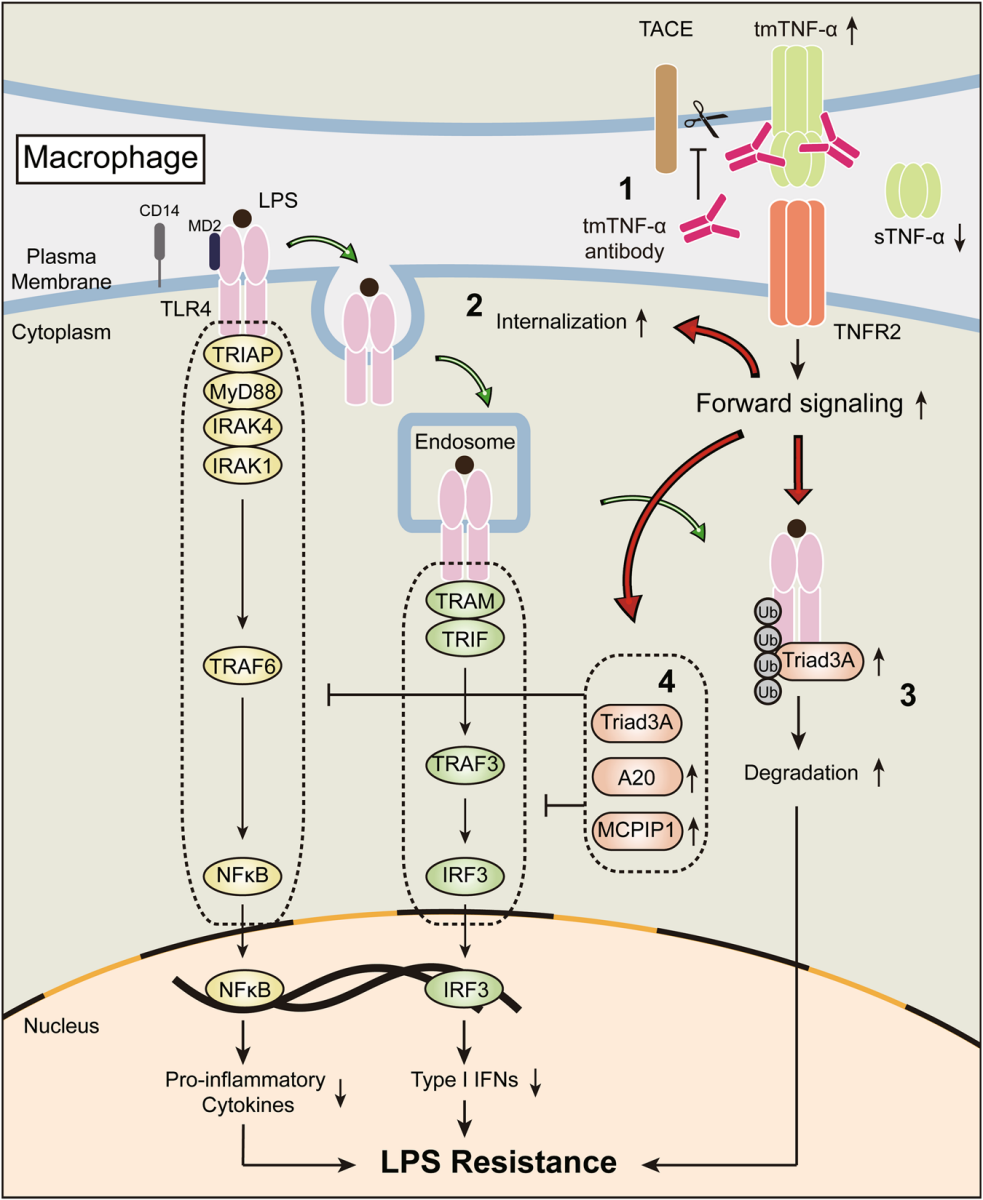
The mechanisms of tmTNF-α Ab-induced LPS resistance. tmTNF-α Ab suppresses ectodomain shedding of tmTNF-α, increasing tmTNF-α expression, and decreasing sTNF-α release and its detrimental effects (1). Increased tmTNF-α expression induced by the Ab results in LPS resistance via TNFR2 by promotion of LPS-induced TLR4 internalization (2) and degradation through recruiting Triad3A to TLR4 to induce its ubiquitination (3), and by upregulation of gene expression of A20 and MCPIP1 to suppress TLR4-mediated activation of MyD-88- and TRIF-dependent signaling pathways (4)

At the postreceptor level, we found that tmTNF-α Ab blocked MyD88- and TRIF-dependent signaling pathways. The tmTNF-α Ab reduced IκBα degradation and phosphorylation of p65, MAPK, and IRF3 in response to LPS, thereby decreasing expression of NF-κB-targeted genes, including iNOS and inflammatory cytokines, as well as expression of IRF3-targeted genes such as IFN-β. These results suggested that increased levels of tmTNF-α promoted by the antibody are responsible for the inhibitory effect on LPS/TLR4 signaling, which was supported by the evidence showing that exogenous tmTNF-α suppressed LPS-induced activation of NF-κB and MAPK through TNFR2. In mechanistic studies, we found that either tmTNF-α Ab or exogenous tmTNF-α upregulated LPS-induced mRNA levels of A20 and MCPIP1 in macrophages. A20, a ubiquitin-modifying enzyme that interferes with sTNF-α-mediated signaling to NF-κB^31^, and MCPIP1 deubiquitinate TRAF6 and negatively regulate NF-κB and JNK signaling to terminate the TLR4 signaling pathway and protect mice from LPS-induced septic shock^32,33^. Our previous study demonstrated that silencing of A20 expression abolishes the suppressive effect of tmTNF-α on NF-κB activation and subsequent production of proinflammatory adipokines^13^. Furthermore, MCPIP1 deubiquitinates TRAF3, which negatively regulates IFN-β expression ^32^. These findings indicated that tmTNF-α actively blocks TLR4 signaling by upregulating expression of these negative regulators in addition to promoting TLR4 internalization and degradation (Fig. 8).

Despite the efficacy of anti-TNF drugs for treatment of rheumatoid arthritis, Crohn’s disease and psoriasis, these drugs block the action of both tmTNF-α and sTNF-α and thus can increase the risk of infection, malignancy and development of secondary autoimmune diseases^34^. Our data demonstrated that tmTNF-α Ab significantly reduced bacterial load in the blood and PLF while exerting anti-inflammatory activity in septic shock. tmTNF-α alone is sufficient to retain a certain level of immunity against pathogens, including resolving infection with Leishmania major in cultured macrophages and in mice^35,36^ and partial protection against acute infection by Myobacterium tuberculosis or Listeria monocytogenes^37–39^. tmTNF-α Ab selectively inhibited detrimental effects of sTNF-α while preserving tmTNF-α-mediated anti-infection and anti-inflammation activities. Thus, for treatment of septic shock and inflammatory diseases, tmTNF-α Ab could be superior to the full blockage of both forms of TNF-α.

## Materials and methods

### Reagents and antibodies

LPS from *Escherichia coli* O111: B4, phorbol myristate acetate (PMA), and MG132 were purchased from Sigma-Aldrich (St. Louis, MO, USA). An antihuman tmTNF-α monoclonal antibody (mAb) was made by our lab^18^ and is unable to cross-react to sTNF-α and murine tmTNF-α. A polyclonal antibody (pAb) specific to the corresponding epitope in murine tmTNF-α was ordered from GL Biochem Ltd (Shanghai, China) and used for murine macrophages and mice models. Isotype IgG (Santa Cruz Biotechnology, Dallas, TX, USA) and normal rabbit serum IgG purified by protein G column (house made) served as controls. The other antibodies used for FACS, immunoprecipitation and Western blotting were listed in Supplementary Table 1.

### Animals and septic shock models

C57BL/6 mice and BALB/c male mice (6–8 weeks) were purchased from Beijing HFK Bioscience Company (Beijing, China). TNFR1 or TNFR2 knockout (KO) mice on a BALB/c background were kindly gifted from Prof. Zhihai Qin (National Laboratory of Biomacromolecules, Institute of Biophysics Chinese Academy of Sciences, Beijing, China). Mice were housed on a 12-h light/12-h dark cycle and cared for in accordance with the National Institutes of Health Guidelines for the Care and Use of Laboratory Animals. The study was approved by the Animal Care and Use Committee of Huazhong University of Science and Technology.

For LPS-induced septic shock, mice were challenged with intraperitoneal injection of 30 mg/kg LPS. Mice were observed for 72 h. For cecal ligation and puncture (CLP)-induced septic shock, a 1 cm midline incision was performed after anaesthetizing mice with ketamine and xylazine. The cecum was exteriorized, ligated at half the distance between distal pole and base, perforated by a single through-and-through puncture with a 21 G needle and extruded a droplet of feces^40^. The sham group underwent the same procedures except CLP. 600 μg of tmTNF-α pAb or the same amount of serum IgG was singly injected intraperitoneally immediately or 5 h after the CLP operation or 30 min before LPS treatment.

### Cell culture and preparation of PBMC, peritoneal macrophages, and BMDM

A human monocyte cell line THP-1 and a murine macrophage cell line Raw264.7 were obtained from American Type Culture Collection (ATCC) and cultured in RPMI-1640 or DMEM supplemented with 10% heat-inactivated fetal calf serum (FCS; Sijiqing, Hangzhou, China), 100 U/mL penicillin and 100 μg/mL streptomycin at 37 °C in 5% CO_2_. THP-1 cells were differentiated into macrophages by stimulation with 100 ng/ml PMA for 3 days. Peripheral human blood mononuclear cells (PBMC) from healthy donors were separated by Ficoll–Paque density gradient centrifugation, followed by adherence for 1 h in 10% FCS RPMI-1640 medium at 37 °C. Peritoneal macrophages were isolated from mice by peritoneal lavage with cold sterile PBS.

Bone marrow-derived macrophages (BMDM) were isolated by flushing the femurs of BALB/c mice with PBS. Cells were cultured in DMEM with 10% heat-inactivated FCS and L929 conditioned medium at a ratio of 2:1. On day 4, the medium was exchanged with fresh medium of the same composition. After 7 days, >95% adherent cells were macrophages as evidenced by F4/80 expression (Becton, Dickinson and Company).

### Mutation, plasmids construction, and transfection

The plasmid pIRES2-EGFP containing human wild-type TNF-α cDNA was constructed in our laboratory as previously described^18^. A plasmid PMD18T-hTACE, encoding full length of human TACE cDNA, was gifted from Dr. Wei Huang (Wuhan Institute of Medical Sciences, Wuhan, China). The ▵M-TACE cDNA, encoding TACE mutant that lacks metalloprotease domain (position from 215 to 473), was generated by overlap PCR using PMD18T-hTACE as a template and two pairs of primers: primer 1 (forward), 5′-GGAATTCATGAGGCAGTCTCTCCTATTCC-3′, and primer 2 (reverse), 5′-TTCCCACAAACTTTATTGCTTCTTTTCACTCGAT-3′; primer 3 (forward), 5′-GCTTGTTCATCGAGTGAAAAGAAGCAATAAAGTTTG-3′ and primer 4 (reverse), 5′-CGGGATCCCCGCACTCTGTTTCTTTGCTGTCA-3′. ▵M-TACE cDNA was inserted into the eukaryotic expression vector pDsRed-monomer-N1 at *Eco*RI and *Bam*HI site. The construct was verified by DNA sequencing (TSINGKE Biological Technology).

HEK 293T cells were transfected or cotransfected with expression plasmids pIRES2-EGFP/wtTNF-α or/and pDsRed-monomer-N1/▵M-TACE using Lipofectamine™ 2000 (Invitrogen, Carlsbad, CA, USA) according to the manufacturer’s instructions. After 24 h post-transfection, cells were stimulated for 4 h with 100 ng/ml LPS, or 60 ng/ml PMA, combined with 2 μg/ml tmTNF-α mAb, 2 μg/ml isotype antibody IgG, or 10 µM TACE inhibitor TAPI-1, respectively. Total or soluble protein was extracted from cells or culture supernatants for western blot analysis.

### Harvesting of exogenous murine and human tmTNF-α

NIH3T3 cells or HEK 293T cells were stably transfected with a retrovirus vector pMCSV-CMV-P2A-puro containing murine wild-type TNF-α cDNA at *Eco*RI and *Bam*HI site (Hanbio, Biotechnology Co., Ltd., Shanghai, China) or cotransfected with a lentiviral vector pTK642 containing human wild-type TNF-α cDNA at *Bam*HI and *Xho*I site, a packaging vector psPAX2 and an envelope vector pMD2G (gifted form Prof. Tongcun Zhang, Wuhan University of Science and Technology, Wuhan, China), respectively. tmTNF-α overexpressing NIH3T3 cells or 293T cells as effector cells were fixed with 4% paraformaldehyde for 30 min at room temperature and were used as the source of exogenous murine or human tmTNF-α, respectively. Murine RAW264.7 and BMDM, and human THP-1-derived macrophages as target cells were coincubated with corresponding effector cells at an effector/target ratio of 10:1.

### Pull-down assay

The cDNA of −23~157 human TNF-α (−23~157 hTNF-α) that contains extracellular domain and part of transmembrane domain of TNF-α was amplified by PCR from pEGFP N1-TNF-α using forward primer (5′- CGCGGATCCATGGGAGTGATCGGCCCCCAG -3′) and reverse primer (5′- CCGCTCGAGTCACAGGGCAATGATCCCAAA -3′), and was cloned into pET-28a vector at *Bam*HI and *Xho* I site. The constructed plasmid containing His-tagged −23~157 hTNF-α (−23~157 TNF-α-His) was transformed and expressed in *E. coli* strain Rosetta. Then, the −23~157 TNF-α-His was purified and immobilized on Ni-NTA-resin. This Ni-NTA-resin was preincubated with 2 μg tmTNF-α mAb or isotype IgG for 4 h at 4 °C, followed by further incubation with total protein from HEK 293T cells (expressing endogenous TACE) overnight at 4 °C with continuous rocking. After washing with PBS, the resin-bound proteins were eluted and analyzed by western blotting.

### Western blot analysis

Total protein was extracted by lysis of cells with ice-cold NP-40 lysis buffer (Beyotime Biotechnology, Shanghai, China) or by the homogenization of liver tissue in RIPA lysis buffer (BOSTER, Wuhan, China) containing protease inhibitors 0.5 mM PMSF, 5 μg/ml aprotinin, and 5 μg/ml leupeptin, followed by incubation on ice for 30 min at 4 °C. After centrifugation at 12,000 × *g* for 15 min at 4 °C, total protein in the supernatant was collected. For soluble protein isolation, 500 μl of culture supernatant was mixed with 500 μl of methanol and 125 μl of chloroform, vortexed and centrifuged at 12,000 × *g* for 10 min at 4 °C. The upper phase was removed and 500 μl of methanol was added and mixed. After centrifugation at 12,000 × *g* for 10 min at 4 °C, the pellet was dissolved in loading buffer for western blotting^41^.

Fifty micrograms of total or soluble protein was separated on a 12% SDS-polyacrylamide gel and transferred to PVDF membrane (Millipore, Billerica, MA, USA) using a semi-dry transfer system (Bio-Rad Laboratories). After blocking with 5% (w/v) nonfat milk in TBS-Tween 20 (0.05%) for 2 h at RT, membranes were incubated overnight at 4 °C with primary antibodies against TNF-α, TACE, TLR4, Triad3A, IκBα, β-actin, phosphorylated p65, p65, phosphorylated IRF3, and IRF3. Then, membranes were incubated with horseradish peroxidase-conjugated secondary antibodies (Feiyi Biotech, Wuhan, China) for 2 h. The bands were visualized with SuperSignal West Pico Chemiluminescence Substrate (Thermo, Waltham, MA, USA).

### Immunoprecipitation (IP)

The total protein extracted after stimulation was pretreated with 1 μg of normal mouse IgG and 25 μl of Protein G PLUS-Agarose (Santa Cruz Biotechnology) at 4 °C for 1 h to remove nonspecific binding, then followed by incubation overnight with a monoclonal antibody specific to TLR4 at 4 °C. Subsequently, the immune complexes were incubated with Protein G PLUS-Agarose in rotation at 4 °C for 4 h. The immunoprecipitated molecules were analyzed by western blotting.

### Quantitative real-time PCR

Total RNA was extracted using the TRIzol reagent (Invitrogen) and reverse-transcribed to cDNA using HiFiScript cDNA Synthesis Kit (CoWinBiotech, Beijing, China) according to the manufacturer’s instructions. The primers were synthesized by Tsingke (Wuhan, China) and their sequences were listed in Supplementary Table 2. Realtime-PCR amplification of cDNA was conducted in 20 μl UltraSYBR Mixture (with ROX) (Beijing CoWin Biotech, Beijing, China) using the CFX Connect Real-Time PCR Detection System (Bio-Rad). The reactions were performed in triplicate as follows: 95 °C for 10 min, followed by 40 cycles of 95 °C for 15 s and 60 °C for 1 min. Results were analyzed using the 2^−▵▵^^Ct^ method and normalized to the corresponding level of GAPDH.

### Flow cytometry

To detect cell surface expression of tmTNF-α and TLR4, cells were incubated at 4 °C for 1 h with PE-conjugated anti-murine TNF-α antibody or primary antibodies including anti-human TNF-α and anti-human TLR4 antibodies, followed by FITC-conjugated secondary antibodies. The fluorescence-stained cells were analyzed on a LSR II flow cytometer (Becton Dickinson, San Jose, CA, USA).

### ELISA for cytokines and NO detection

The concentrations of sTNF-α, IL-1β, IL-6, IL-10, IFN-β and sTNFR1 were determined using commercial ELISA kits according to the manufacturer’s instructions. ELISA kits for sTNF-α, IL-1β, IL-6 and IL-10 were purchased from eBioscience (San Diego, CA, USA). IFN-β ELISA Kit or sTNFR1 ELISA Kit was from Signalway Antibody LLC (Maryland, USA) or USCN Life Science Inc. (Houston, TX, USA), respectively.

NO was detected by a commercial Nitric Oxide Assay Kit (Beyotime Biotechnology, Shanghai, China) according to the manufacturer’s instructions.

### Generation of Cas9-CRISPR-mediated TNF-α knockout in THP-1 cells

The Cas9-CRISPR system was used to generate TNF-α gene knockout (KO) in THP-1 cells and targeting sequence (TGAAAGCATGATCCGGGACG) was designed using the web-based tool CRISPR Design at crispr.mit.edu. A lentivirus plasmid GV393 containing TNF-α-targeted guide RNA, Cas9 and EGFP was ordered from Shanghai Genechem Co., Ltd. THP-1 cells were infected with virus (50 MOI). At 48 h after infection, GFP-positive cells were sorted by flow cytometry (Beckman MoFlo) to allow single-colony formation. After 14 days, individual colonies were picked and selected clones were expanded. The absence of TNF-α was confirmed by western blotting.

### TNF-α bioassay

TNF-α-KO THP-1 cells were stimulated with 100 ng/ml LPS and 2 μg/ml human tmTNF-α mAb or 10 µM TAPI-1 for 12 h and the culture supernatants were collected. L929 cells were seeded in a 96-well plate and incubated overnight. 10 ng/ml sTNF-α (PeproTech company, Rocky Hill, NJ, USA) and 100 μl of the culture supernatant were added and incubated at 37 °C for 12 h in the presence of 1 μg/ml actinomycin D (Sigma-Aldrich). Cell viability was then measured by staining for 4 h with PBS containing 0.5 mg/ml MTT (Sigma-Aldrich). The OD values were detected at 570 nm on a microplate reader (Tecan, Groedig, Austria). The cytotoxicity of sTNF-α was calculated by the following formula: Cytotoxicity (%) = (1 − OD_sample_/OD_control_) × 100%.

### Bacterial load

Peripheral blood and peritoneal lavage fluid were aseptically collected at 24 h after the CLP operation. Samples were serially diluted with sterile saline and cultured overnight at 37 °C on tryptic soy agar plates. The number of bacterial colonies was counted in a blind manner and expressed as colony forming units (CFU) per milliliter.

### Statistical analysis

Statistical analysis was performed with GraphPad Prism V6 software using one-way ANOVA followed by post hoc Turkey’s test. The survival curves were plotted using the Kaplan–Meier method and compared by the log-rank test. A value of *P* < 0.05 was considered statistically significant.

## Supplementary information

Supplementary data-clean version.
