# Supplementary material for: Inhibition of transmembrane TNF-α shedding by a specific antibody protects against septic shock
Source: Cell Death Dis. 2019 Aug 5;10(8):586. doi: 10.1038/s41419-019-1808-6 (PMC6683172; doi:10.1038/s41419-019-1808-6)
Supplement: Supplementary file 1 — Supplementary data-clean version. [file 41419_2019_1808_MOESM1_ESM.docx]

**Supplementary Data**

**Materials and Methods：**

**Supplementary Table 1．****Primary antibodies used for flow cytometry, IP and Western blotting**

| **Name of antibody** | **Catalog number** | **Company** | **Application** |
| --- | --- | --- | --- |
| rabbit anti-human sTNF-α |  | House made | WB, FCM |
| anti-TACE | 1131 | ProSci | WB |
| anti-TLR4 | sc-10741 | Santa Cruz Biotechnology | WB |
| anti-Triad3A | 3371 | ProSci | WB |
| anti-IκBα | sc-371 | Santa Cruz Biotechnology | WB |
| anti-β-actin | AC026 | ABclonal | WB |
| anti-p65 | 14-6731-81 | Invitrogen | WB |
| anti-p-p65 | 3033 | Cell Signaling Technology | WB |
| anti-IRF3 | 11312-1-AP | Proteintech | WB |
| anti-p-IRF3 | 29047 | Cell Signaling Technology | WB |
| anti-ERK | sc-514302 | Santa Cruz Biotechnology | WB |
| anti-p-ERK | 4370 | Cell Signaling Technology | WB |
| anti-JNK | sc-7345 | Santa Cruz Biotechnology | WB |
| anti-p-JNK | 4668 | Cell Signaling Technology | WB |
| anti-p38 | sc-7972 | Santa Cruz Biotechnology | WB |
| anti-p-p38 | 4511 | Cell Signaling Technology | WB |
| anti-TNFR1 | sc-8436 | Santa Cruz Biotechnology | WB |
| PE-conjugated anti-mouse TNF-α | 12-7321-81 | eBioscience | FCM |
| anti-human TLR4 | 551964 | BD Biosciences | FCM |
| anti-TLR4 | sc-293072 | Santa Cruz Biotechnology | IP |

FCM: Flow cytometry; WB: Western blot; IP: Immunoprecipitation

**Supplementary Table 2．****The sequences of primers for real-time PCR**

| **Gene** | **Species** | **Primers** |
| --- | --- | --- |
| IL-1β | Human | F: 5’- TTCGACACATGGGATAACGAGG -3’ |
|  |  | R: 5’- TTTTTGCTGTGAGTCCCGGAG -3’ |
| IL-6 | Human | F: 5’- GGATTCAATGAGGAGACTTGC -3’ |
|  |  | R: 5’- GTTGGGTCAGGGGTGGTTAT -3’ |
| IL-10 | Human | F: 5’- CCCAAGCTGAGAACCAAGAC -3’ |
|  |  | R: 5’- AAGGCATTCTTCACCTGCTC -3’ |
| TLR4 | Human | F: 5’- GACCTGTCCCTGAACCCTATGA -3’ |
|  |  | R: 5’- TTCTAAACCAGCCAGACCTTGA -3’ |
| iNOS | Human | F: 5’- AAGCCCAAGGTCTATGTTCAGG -3’ |
|  |  | R: 5’- TCGTAAGGAAATACAGCACCAAAG -3’ |
| A20 | Human | F: 5’- AACATTTTGCTGCTGCCTC -3’ |
|  |  | R: 5’- AGGTGCTTTGTGTGGTTCG -3’ |
| MCPIP1 | Human | F: 5’- GCAGGCCAGCGTGTATACTAAGC -3’ |
|  |  | R: 5’- TGCTGGGACTTGTAGGAGAGGA -3’ |
| IFN-β | Human | F: 5’- CAGTCACTGTGCCTGGACCATA -3’ |
|  |  | R: 5’- GTTTCGGAGGTAACCTGTAAGTCTG -3’ |
| GAPDH | Human | F: 5’- CAGTCCATGCCATCACTGCCACCCAG -3’ |
|  |  | R: 5’- CAGTGTAGCCCAGGATGCCCTTGAG -3’ |
| IRAK-M | Human | F: 5’- TGCCTTGGTAGGATTGGGC -3’ |
|  |  | R: 5’- TGTTTACTGCTGCTGCTGGT -3’ |
| SHIP1 | Human | F: 5’- AAGTGTCGTGTCTCCACCC -3’ |
|  |  | R: 5’- CGGGGATTCTCGTTTGAAAAAGG -3’ |
| SOCS1 | Human | F: 5’- GTAGCACACAACCAGGTGGCA -3’ |
|  |  | R: 5’- TCGAAGAGGCAGTCGAAGCTC -3’ |
| SOCS3 | Human | F: 5’- ATGGTCACCCACAGCAAGTTT -3’ |
|  |  | R: 5’- TCACACTGGATGCGCAGGTTC -3’ |
| DUBA | Human | F: 5’- ACCAGGTGTATGGAGACCAGGAC -3’ |
|  |  | R: 5’- ATGGGTTCGTCCTCGTTTTGA -3’ |
| Pin1 | Human | F: 5’- GTGCCTTCAGCAGAGGTCAGA -3’ |
|  |  | R: 5’- CAGTGCGGAGGATGATGTGG -3’ |
| SHP-2 | Human | F: 5’- AGGATTGAAGAAGAGCAGAAAAGC -3’ |
|  |  | R: 5’- CCACGTTTTCATAGACTCTAGCACTG -3’ |
| IL-1β | Mouse | F: 5’- GCCACCTTTTGACAGTGATGAG -3’ |
|  |  | R: 5’- TGCTGCGAGATTTGAAGCTG -3’ |
| IL-6 | Mouse | F: 5’- AGCCAGAGTCCTTCAGAGAGAT -3’ |
|  |  | R: 5’- AGGAGAGCATTGGAAATTGGGG -3’ |
| IL-10 | Mouse | F: 5’- AGGCGCTGTCATCGATTTCT -3’ |
|  |  | R: 5’- ATGGCCTTGTAGACACCTTGG -3’ |
| TLR4 | Mouse | F: 5’- ACACCACCTCTCAAACTT -3’ |
|  |  | R: 5’- TTGTCTCCACAGCCACCA -3’ |
| iNOS | Mouse | F: 5’- GCTGCCAGGGTCACAACTT -3’ |
|  |  | R: 5’- AACAGCTCAGTCCCTTCACC -3’ |
| A20 | Mouse | F: 5’- ACCATGCACCGATACACGCT -3’ |
|  |  | R: 5’- AGCCACGAGCTTCCTGACTTC -3’ |
| MCPIP1 | Mouse | F: 5’- TGAGACCTGTGGTCATCGACG -3’ |
|  |  | R: 5’- TAGTTCCCGAAGGATGTGCTG -3’ |
| IFN-β | Mouse | F: 5’- TGGGTGGAATGAGACTATTGTTGT -3’ |
|  |  | R: 5’- AAGTGGAGAGCAGTTGAGGACATC -3’ |
| GAPDH | Mouse | F: 5’- TGTGTCCGTCGTGGATCTGA -3’ |
|  |  | R: 5’- TTGCTGTTGAAGTCGCAGGAG -3’ |
| IRAK-M | Mouse | F: 5’- AATGCAGCCAGTCTGAGGTCAC -3’ |
|  |  | R: 5’- GACGAGCCCAAACTGATCACA -3’ |
| SHIP1 | Mouse | F: 5’- ATCTGGGAAAGGTGGAAGCTCT -3’ |
|  |  | R: 5’- GAAGGAACTCACGGATCCATACA -3’ |
| SOCS1 | Mouse | F: 5’- AGACCTTCGACTGCCTTTT -3’ |
|  |  | R: 5’- GGGAAGGAACTCAGGTAGTCACG -3’ |
| SOCS3 | Mouse | F: 5’- CCTCAAGACCTTCAGCTCCAAA -3’ |
|  |  | R: 5’- TCACACTGGATGCGTAGGTTCTT -3’ |
| DUBA | Mouse | F: 5’- CCACATGCAGTTCAGCCACA -3’ |
|  |  | R: 5’- CCTGCTGAGAACTGACTGCTTGT -3’ |
| Pin1 | Mouse | F: 5’- CGCTGCTCACATCTGCTGGT -3’ |
|  |  | R: 5’- GCAAACGACGCATCCTCAAA -3’ |
| SHP-2 | Mouse | F: 5’- CATTGTGATTGACATCCTTATTGACA -3’ |
|  |  | R: 5’- CGCTGCAGCGTCTCTATGTAGT -3’ |

F: Forward; R: Reverse

**Supplementary Figures：**


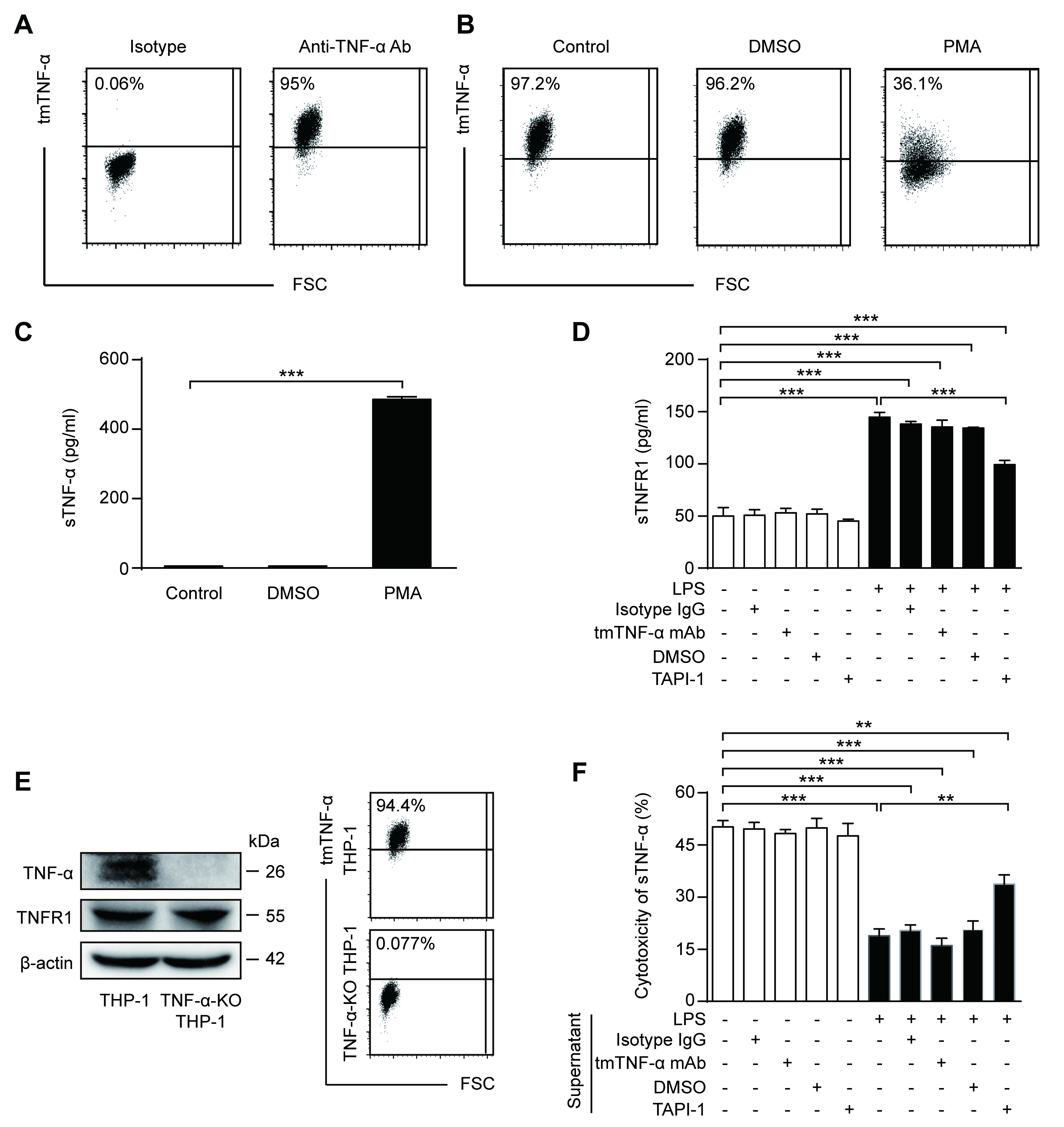


**Supplementary Figure 1：tmTNF-α shedding by PMA stimulation and** **affection of tmTNF-α Ab on LPS-induced sTNFR shedding. (A)** tmTNF-α expression on the cell surface of THP-1 detected by flow cytometry. **(B, C)** THP-1 cells were stimulated by 100 ng/ml PMA for 72 h. DMSO served as a control. tmTNF-α expression on the cells surface was detected by flow cytometry and concentrations of sTNF-α in supernatants were determined by ELISA. **(D)** THP-1 cells were stimulated with 100 ng/ml LPS, combined with 2 μg/ml tmTNF-α mAb, isotype IgG or 10 μM TAPI-1 for 12 h. sTNFR1 concentrations in supernatants were measured by ELISA. (**E**) The efficiency of TNF-α knockdown in THP-1 cells by CRISPR/Cas9 was confirmed by western blot and flow cytometry. (**F**) TNF-α-KO THP-1 cells were stimulated as described in (D) and sTNFR containing culture supernatants were collected to neutralize sTNF-α. The cytotoxicity of sTNF-α to L929 cells was detected by bioassay. All quantitative data are presented as means ± SEM of at least three independent experiments. ***p*<0.01, ****p*<0.001.


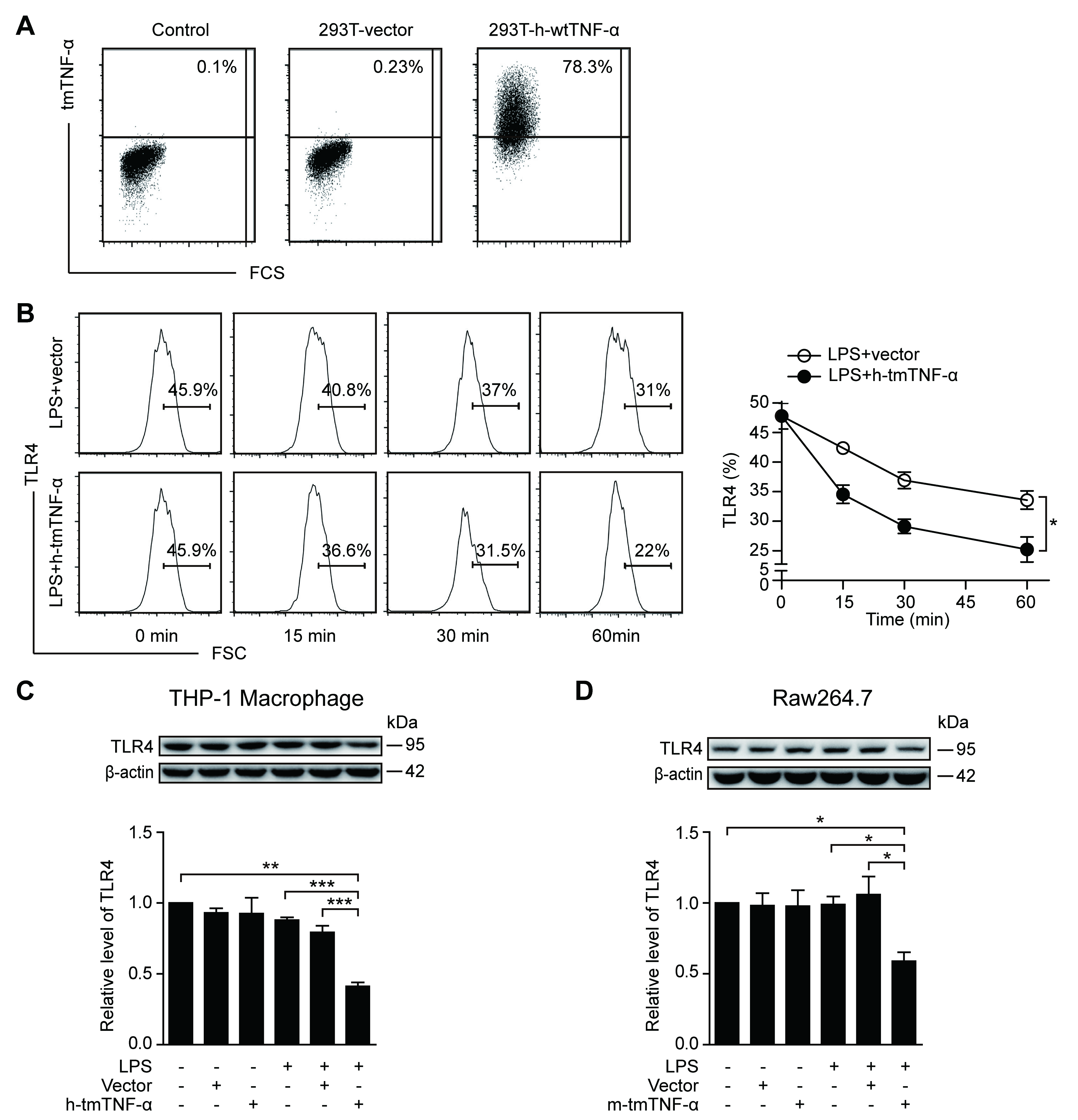


**Supplementary Figure 2.** **Exogenous tmTNF-α facilitates LPS-induced TLR4 internalization and degradation.** **(A)** HEK 293T cells were stably transfected with human TNF-α. Ectopical expression of tmTNF-α on the cell surface was detected by flow cytometry. **(B-D)** Human (h) or murine (m) tmTNF-α on 4% paraformaldehyde-fixed 293T cells or NIH3T3 cells was co-cultured with THP-1-derived or Raw264.7 macrophages at an effector/target ratio of 10:1 for 30 min, followed by 100 ng/ml LPS stimulation. The empty vector transfected cells served as a control. **(B)** TLR4 expression on the cell surface was evaluated by flow cytometry for indicated time points. Representative images of FCM on the left and quantitative data on the right. TLR4 production in THP-1-derived **(C)** and Raw264.7 **(D)** macrophages was detected at 12 h after LPS stimulation. Representative images of western blot analysis from three independent experiments for TLR4 expression (upper) and their quantitative data (lower). All quantitative data are presented as means ± SEM of at least three independent experiments. **p*<0.05, ***p*<0.01, ****p*<0.001.


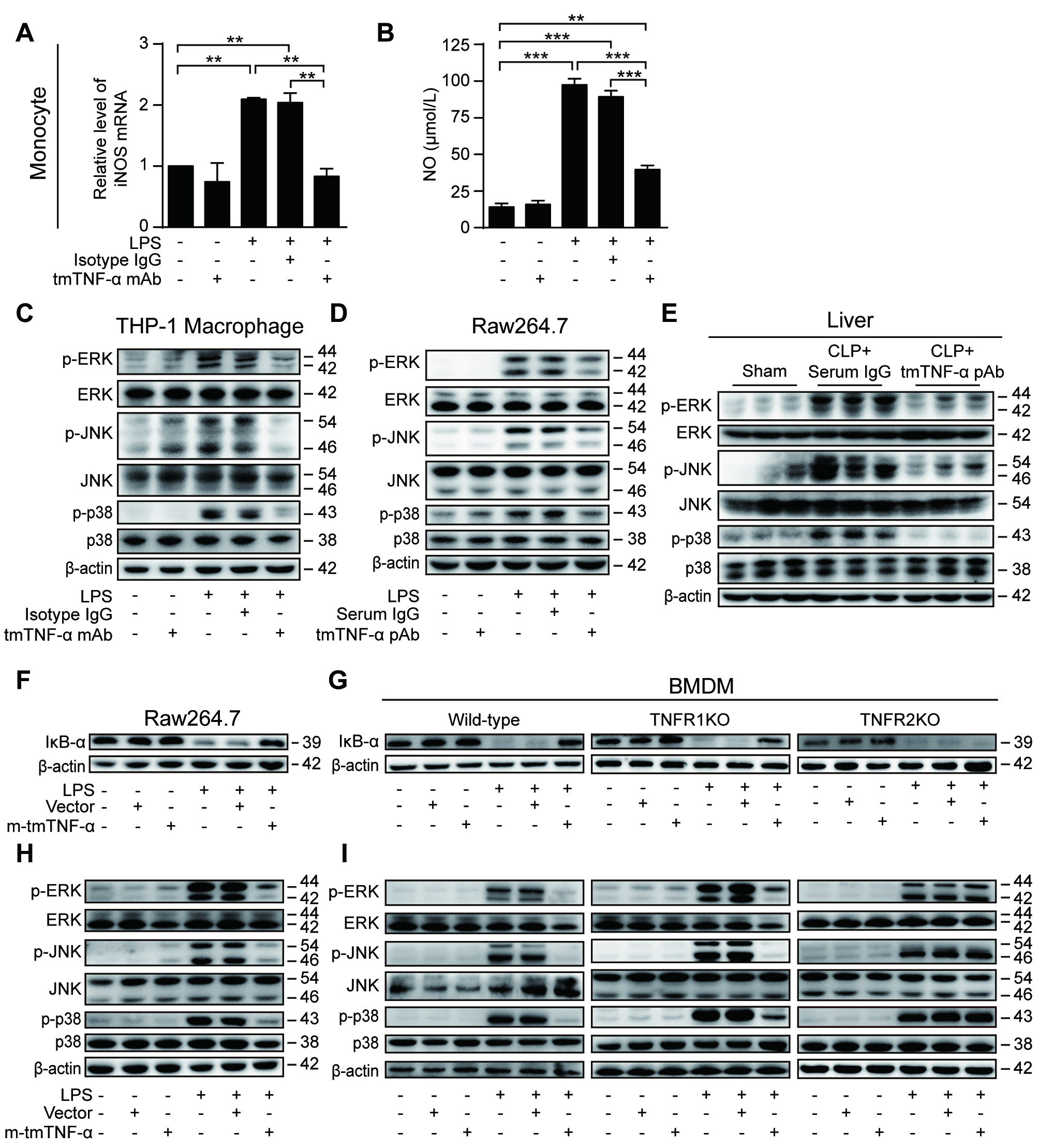


**Supplementary Figure 3. Inhibitory effect of tmTNF-α Ab and exogenous tmTNF-α on LPS-induced MAPK and NF-κB signaling pathways.** **(A, B)** Primary human monocytes were stimulated with 100 ng/ml LPS, combined with 2 μg/ml tmTNF-α mAb or isotype IgG. iNOS mRNA levels were assessed by real-time PCR at 4 h and NO production was measured at 10 h after stimulation. The data are presented as means ± SEM of at least three independent experiments. ***p*<0.01, ****p*<0.001. **(C, D)** THP-1-derived and RAW264.7 macrophages were stimulated for 1 h with 100 ng/ml LPS, combined with 2 μg/ml tmTNF-α mAb or pAb. Isotype IgG or normal serum IgG served as a control. Representative images of western blot analysis from three independent experiments for phosphorylation of ERK, JNK and p38. **(E)** Mice were intraperitoneally injected with 600 μg tmTNF-α pAb or normal serum IgG immediately after the CLP operation. Western blot analysis for phosphorylation of ERK, JNK and p38 in the liver at 24 h after the CLP operation. **(F-I)** Raw264.7 or BMDMs from wild type, TNFR1KO or TNFR2KO BALB/c mice were cultured with murine tmTNF-α on 4% paraformaldehyde-fixed NIH3T3-wtTNF-α cells at an effector/target ratio of 10:1 for 30 min, followed by 100 ng/ml LPS stimulation for another 30 min. Representative images of western blot analysis from three independent experiments for IκBα degradation **(F, G)** and phosphorylation of ERK, JNK and p38 **(H, I)**.


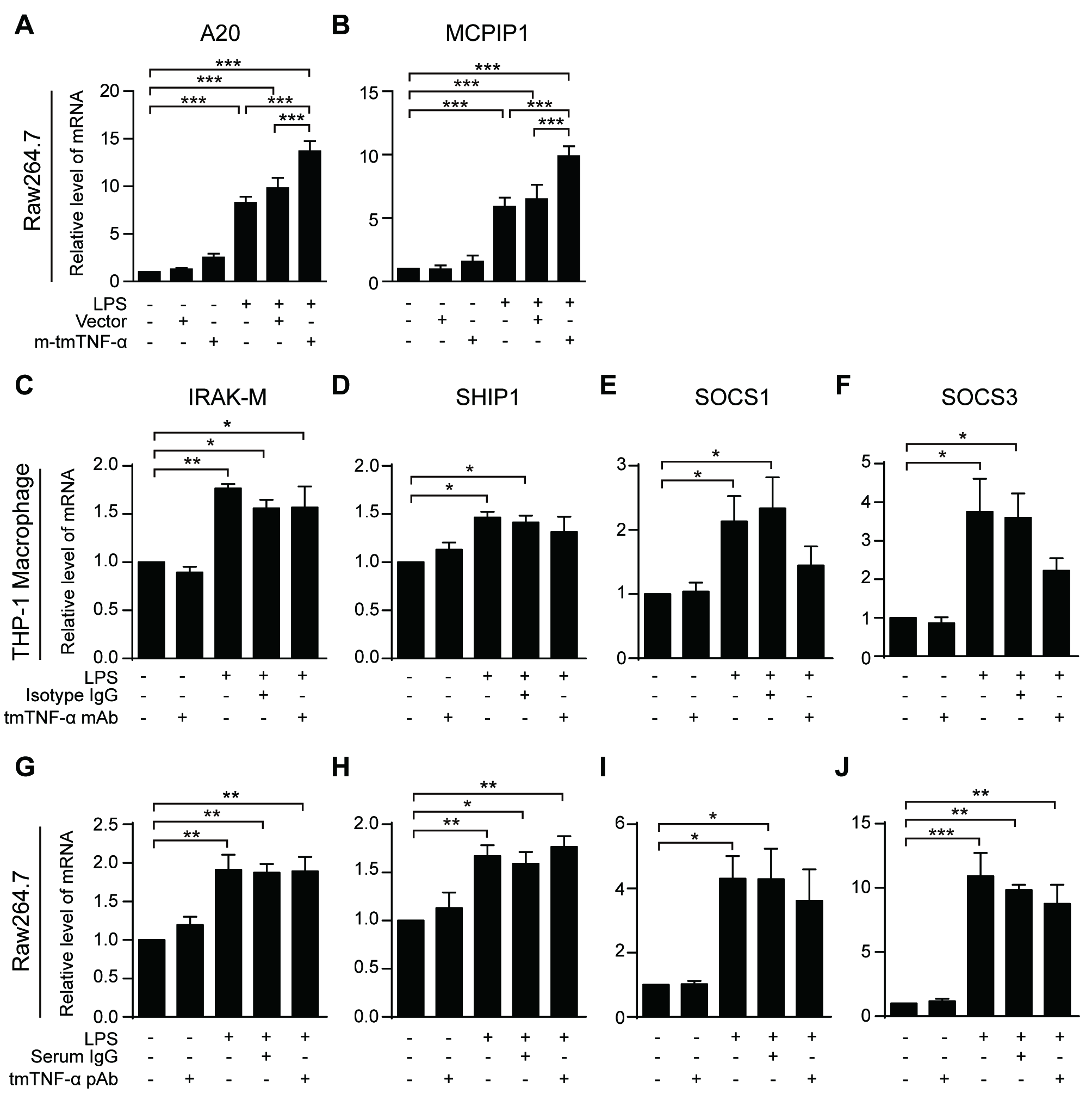


**Supplementary Figure 4. Effect of tmTNF****-α Ab and exogenous tmTNF-α on mRNA levels of negative regulators for TLR4 signaling.** **(A, B)** Raw264.7 macrophages were cultured with murine tmTNF-α on 4% paraformaldehyde-fixed NIH3T3-wtTNF-α cells at an effector/target ratio of 10:1 for 30 min, followed by 100 ng/ml LPS stimulation for 4 h. A20 and MCPIP1 mRNA levels were detected by real-time PCR. **(C-J)** THP-1-derived and RAW264.7 macrophages were stimulated for 4 h with 100 ng/ml LPS, combined with 2 μg/ml tmTNF-α mAb or pAb. Isotype IgG or normal serum IgG served as a control. The mRNA levels of IRAK-M (**C, G**), SHIP1 (**D, H**), SOCS1 (**E, I**) and SOCS3 (**F, J**) were assessed by real-time PCR. All quantitative data are presented as means ± SEM of at least three independent experiments. **p<0.05,* ***p*<0.01, ****p*<0.001


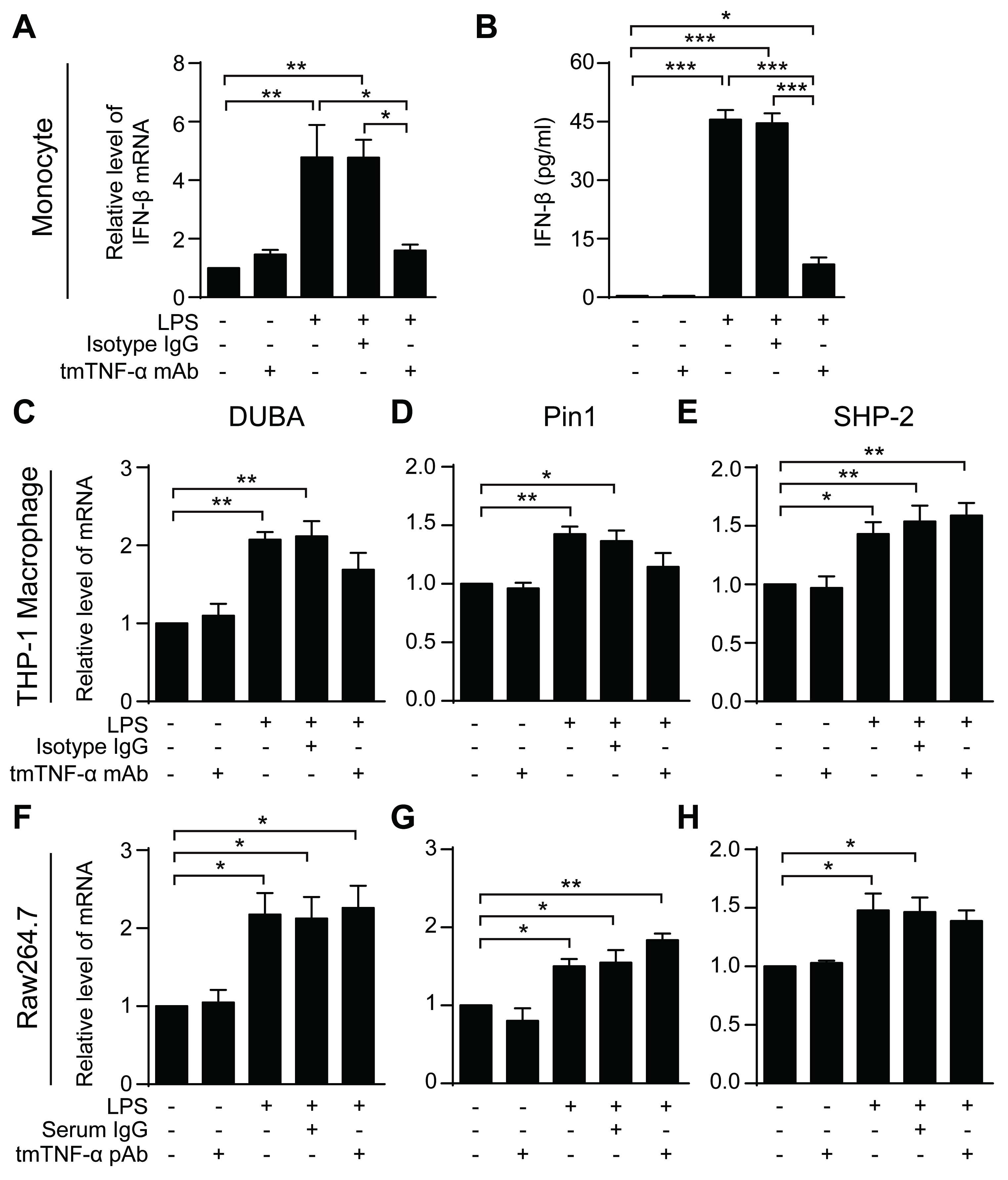


**Supplementary Figure 5.** **Effect of tmTNF-α Ab on mRNA levels of DUBA, Pin1 and SHP-2. (A, B)** Primary human monocytes were stimulated with 100 ng/ml LPS, combined with 2 μg/ml tmTNF-α mAb or isotype IgG. IFN-β mRNA levels were assessed by real-time PCR at 4 h and IFN-β production was measured at 10 h after LPS stimulation. THP-1-derived and Raw264.7 macrophages were stimulated with 100 ng/ml LPS, combined with 2 μg/ml tmTNF-α mAb or pAb for 4 h. Isotype IgG or normal serum IgG served as a control. The mRNA levels of DUBA (**C, F**), Pin1 (**D, G**) and SHP-2 (**E, H**) were assessed by real-time PCR. All quantitative data are presented as means ± SEM of at least three independent experiments. **p*<0.05, ***p*<0.01, ****p*<0.001.
